# Evidence for Causal Links Between Known Modifiable Risk Factors and Dementia: A Systematic Review of Mendelian Randomisation Studies

**DOI:** 10.1111/ene.70458

**Published:** 2025-12-04

**Authors:** Roopal Desai, Amber John, Emma Anderson, Jean Stafford, Aysha Mohamed Rafik Patel, Natalie L. Marchant, Georgina Charlesworth, Verena Zuber, Joshua Stott

**Affiliations:** ^1^ ADAPT Lab, Research Department of Clinical Educational and Health Psychology University College London London UK; ^2^ Division of Psychiatry University College London London UK; ^3^ MRC Unit for Lifelong Health and Ageing at UCL London UK; ^4^ Department of Epidemiology and Biostatistics Imperial College London London UK; ^5^ MRC Centre for Environment and Health at Imperial College Imperial College London UK

**Keywords:** dementia, mendelian randomisation, modifiable risk factors, systematic literature review

## Abstract

**Background:**

We aimed to systematically review the evidence for associations between the known modifiable risk factors and dementia based on Mendelian randomisation (MR) studies.

**Method:**

Five databases were searched from inception to April 2024 investigating the association between the 12 risk factors identified in the Lancet Commission and dementia. Evaluable analyses were categorized into one of four levels (robust, probable, suggestive, insufficient) based on estimate significance level and concordance of direction of effect between main and sensitivity analyses. Evidence from clinically diagnosed dementia outcomes was synthesized separately from proxy outcomes. A post hoc sensitivity analysis excluded estimates with concerns over construct validity.

**Results:**

A total of 47 studies were included, representing 240 MR associations (185 unique and evaluable). Over half (73.5%) of evaluable analyses were graded as providing insufficient evidence for a causal association. Among clinically diagnosed outcomes, the strongest evidence was for educational attainment (mainly probable evidence in a protective direction) and type 2 diabetes‐related dysfunction (probable evidence in the risk direction). Smoking showed probable evidence of a protective association. Other risk factors, produced inconclusive or insufficient evidence. Proxy outcome analyses yielded weaker findings; in particular, the association between education and Alzheimer's disease reversed direction.

**Conclusion:**

MR evidence for most Lancet Commission risk factors remains insufficient or inconclusive. The most consistent support for causal associations was observed for lower educational attainment and type 2 diabetes. Null findings should be interpreted cautiously given limitations in GWAS phenotyping, sample composition, and MR methodology.

## 1 Introduction

Dementia is a clinical syndrome, most often caused by neurodegenerative diseases such as Alzheimer's disease and vascular dementia, which typically affect people aged 65 and over. Although dementia is not an inevitable consequence of ageing, age is the strongest predictor for dementia and as lifespans increase across the globe so will the number of people living with dementia. In 2019 the number of people globally living with dementia was estimated to be in excess of 55 million [1] and this figure is forecast to rise to an estimated figure of 79 million by 2030 and further increase to 139 million by 2050 [2]. The cost of dementia is great and is borne formally through the state and informally by individuals affected and their carers. Taking both these costs together the global cost of dementia was estimated as 1.3 trillion USD in 2019 [1]. To date the progress on developing effective pharmaceutical interventions to halt or diminish the disease has been slow and as a result research has increasingly expanded to identifying modifiable life‐style risk factors for dementia [3].

The Lancet Commission report on dementia prevention, intervention and care [3, 4] extensively reviewed the evidence on modifiable risk factors for dementia. The authors identified nine key risk factors, divided into three life stage categories: early (age < 45 years), mid (age 45–65) and late (age > 65). Less education was found to be an early life risk factor for dementia, with hearing loss, hypertension and obesity representing mid‐life risks, and late‐life factors including smoking, depression, physical inactivity, social isolation and diabetes. These nine risk factors were further extended in 2020 [3] to 12 by inclusion of the mid‐life risk factor of excessive alcohol consumption, traumatic brain injury and the late‐life risk factor of air pollution.

For each risk factor the Lancet Commission calculated the population attributable fraction (PAF), a statistic used to estimate the proportion of disease cases in a population that can be attributed to the specific risk factor. Combining the PAFs for all 12 risk factors, the overall PAF was estimated to be 40%. In other words, findings from the Lancet Commission have been interpreted as meaning that elimination of 12 of the risk factors would result in a reduction of 40% of incident dementia. However, this interpretation is based on a key assumption in the PAF calculation of a *causal relationship* between the risk factor and the outcome. Given that the majority of the evidence in the Lancet Commission report came from observational studies, it is not possible to infer causation.

One approach to building evidence for causality in epidemiology is through triangulating results using different approaches [5]. Triangulation is the idea that different methodological approaches are vulnerable to distinct sources of biases. If two or more approaches aimed at answering the same question yield similar associations, then the evidence of a causal link is strengthened. Mendelian randomisation (MR) is a method that has emerged in recent years as a powerful tool for identifying causal relationships between modifiable risk factors and diseases [6]. MR uses genetic variants as instrumental variables to test the causal effect of an exposure on an outcome. This may be particularly valuable in dementia research, where the long prodromal phase of Alzheimer's disease means that even longitudinal observational studies spanning a decade or more can remain vulnerable to reverse causation. This approach takes advantage of the fact that genetic variants are randomly allocated at conception and are therefore less susceptible to the confounding and reverse causation biases common in observational studies. MR relies on a different set of assumptions compared to traditional observational studies. While it can mitigate confounding and reverse causation, MR results may still be affected by issues such as weak instruments, horizontal pleiotropy, or inadequate instrument validity. Some concerns raised, such as misclassified outcomes, exposure construct validity, and survivor bias, are not unique to MR and also apply to other non‐randomised study designs [5].

In recent years, several MR studies have been conducted to investigate the relationship between modifiable risk factors and dementia. These studies have provided valuable insights into the potential modifiable risk factors for dementia and the potential impact of interventions targeting these risk factors. This systematic review aims to synthesize the findings of these MR studies and provide an overview of the evidence for links between known modifiable risk factors and dementia using MR.

## 2 Methods

### 2.1 Systematic Search and Study Selection

The protocol for the current review was completed in advance and registered on PROSPERO (CRD42021254793). Five databases were searched from inception to April 2024. These databases were: Medline, Embase, PsycINFO, PubMed and Web of Science. Search terms relevant to MR (Mendelian randomisation OR instrumental variable OR genetic instrument OR causal inference) were combined with search terms relevant to cognition or dementia (Alzheimer* OR dement* OR cognit* OR neurocognit* OR memory OR vascular dementia OR mild cognitive impairment OR MCI OR cognitive dysfunction OR cognition change OR frontotemporal dementia OR Lewy body dementia). After de‐duplication, the remaining articles were subject to a title and abstract screen to identify relevant articles for full‐text inspection. Articles were assessed for inclusion based on predetermined inclusion and exclusion criteria. Articles were included if they were: MR studies assessing the association between a modifiable risk factor and dementia or dementia‐related outcomes (e.g., age of onset, proxy risk); the modifiable risk factor was one of the 12 risk factors for dementia as identified by the Lancet Commission on Dementia [3]; available in English; and peer‐reviewed. Articles were excluded if they were: non‐genetic studies or genetic studies other than MR; studies with outcomes that were not directly dementia‐related (e.g., biomarker studies); review articles; and animal studies. Relevant reviews and all studies identified for inclusion were further subject to forward (citation searching) and backward searching (hand searching reference lists). One reviewer (RD) completed the primary search, title and abstract screen and full‐text screen, and a second reviewer (AJ) carried out an independent title and abstract and full‐text screen on 10% of the hit results at each stage. Inter‐rater reliability was calculated for each stage of the screening process. Disagreements were resolved through discussion in consensus meetings and inter‐rater reliability was calculated for each stage of the screening process.

### 2.2 Data Extraction

Two reviewers (RD and AJ) independently extracted all the data (Table S1), including information on exposure and outcome, the genetic instrument, number of single nucleotide polymorphisms (SNP) *F*‐statistic, the genome‐wide association study (GWAS) datasets used in the analysis, MR design (i.e., one‐sample or two‐sample), the main MR estimate as reported by the study author and additional estimates obtained as part of sensitivity analyses.

### 2.3 Evaluation of Evidence

Each MR estimate was evaluated for robustness of evidence for causality based on an established framework [7]. One reviewer (RD) completed the evaluation on all estimates and another reviewer (EA) independently evaluated 20% of the estimates with disagreements resolved through discussion. As the evaluation was based on assessment of both the main analysis and at least one additional sensitivity analysis (e.g., MR Egger) if a study did not report any MR estimates in addition to their main finding, the estimate was categorised as ‘non‐evaluable’. Where an estimate was deemed to be evaluable it was further categorised into one of four levels: ‘robust’, ‘probable’, ‘suggestive’, and ‘insufficient’.

*Robust genetic evidence* for causality was defined as the main MR estimate and all additional sensitivity estimates reported as significant, with concordant directions of effect. Where study authors applied a correction for multiple testing, we adopted their reported significance threshold. In cases where no correction was applied, or a threshold of 0.05 was used despite multiple comparisons, we recalculated significance using the Benjamini–Hochberg false discovery rate method (*q* = 0.05).

*Probable genetic evidence* for causality was defined as at least one method, either the main or a sensitivity estimate, being significant with concordant directions of effect.

*Suggestive genetic evidence* for causality was defined as at least one method, either the main or a sensitivity estimate being significant, with non‐concordant directions of effect. In addition, in the situation where no sensitivity analysis was reported but the main MR result was reported as significant this was evaluated as ‘suggestive’ rather than ‘non‐evaluable’. This was based on the conservative premise that had sensitivity analyses been conducted and the reported estimates been non‐significant and non‐concordant then the estimate would have been evaluated as ‘suggestive’.

*Insufficient genetic evidence* for causality was defined as all estimates being non‐significant or the instrumental variable being deemed not valid.

The number of estimates falling into each category of robustness evaluation was summed to create an aggregated value. To aggregate these values, only unique combinations of exposure and outcome datasets were included. That is, as the majority of GWAS datasets used in MR studies are publicly available, multiple study authors had conducted similar analyses using the same exposure and outcome GWAS datasets. Where multiple studies used the same exposure and outcome dataset combination, only one estimate was included in the aggregated results. In this situation, the included estimate was the one deemed to be evaluable. Where multiple analyses were evaluable then the study estimate included was the one with the highest quality rating.

Given emerging concerns about potential biases introduced by proxy phenotypes in Alzheimer's disease GWAS‐by‐proxy (GWAX) designs, including survivor bias and non‐random survey participation [8] we synthesised the evidence separately for studies that used clinically diagnosed dementia outcomes and those using proxy outcomes based on family history.

### 2.4 Quality Assessment

A quality assessment on all studies using a quality scoring system as proposed by Treur et al. [9]. This scoring system rates the quality of important aspects of MR studies including phenotype measurement (sample size and quality of the exposure and outcome measurements), instrument strength (*p*‐value threshold, number of SNPs, biological knowledge, *F*‐statistic and % variance that the instrument explains). Each aspect is given a quality rating which can be interpreted as ‘low,’ ‘moderate,’ and ‘high’. An overall rating was achieved by consideration of key indicators. Two study authors (RD and J Stafford) independently rated each study with disagreements resolved in consensus meetings. Studies given a ‘low’ rating were removed before the synthesis.

## 3 Results

### 3.1 Study Selection

**FIGURE 1:**
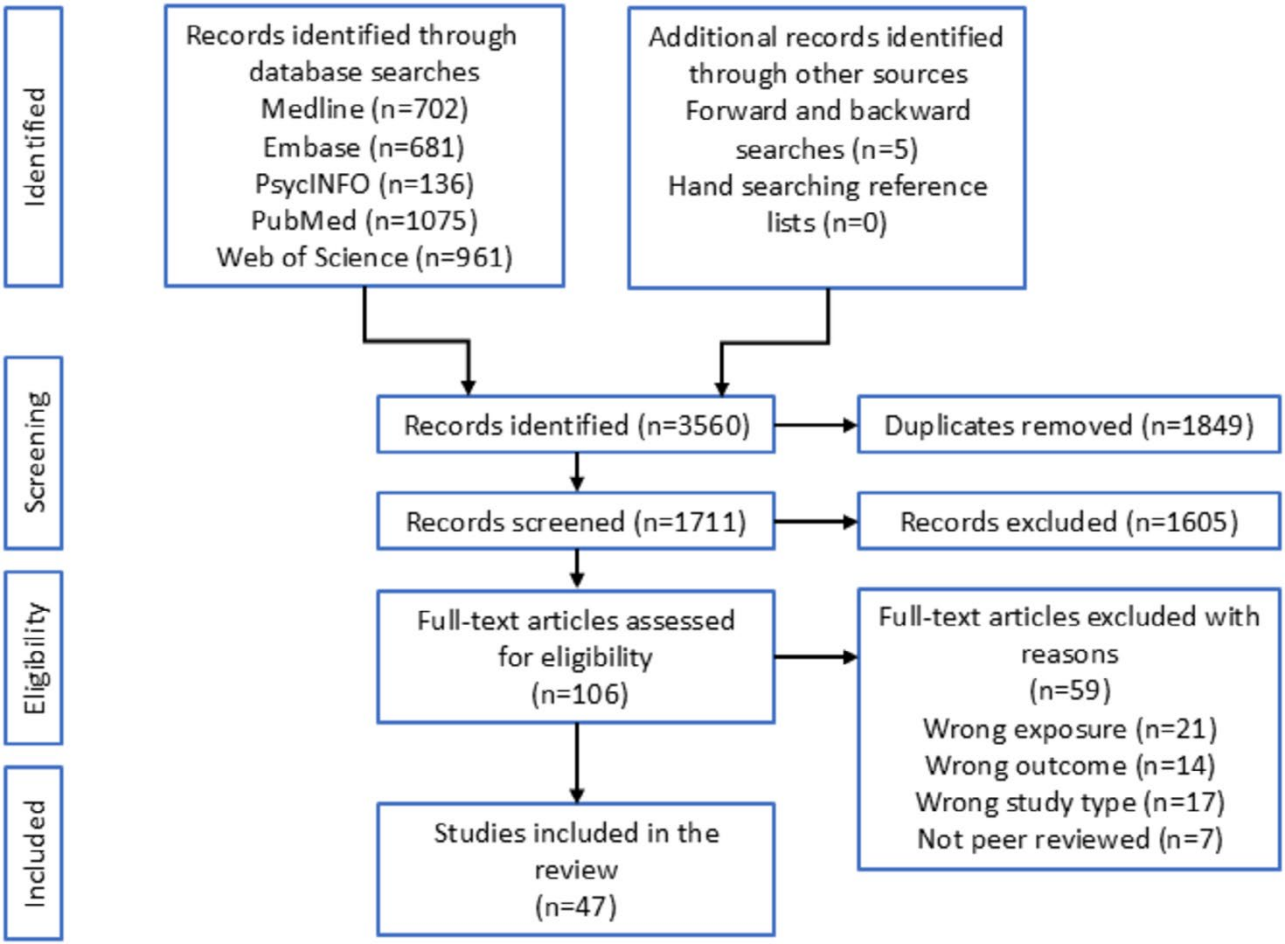
PRISMA Flow diagram of study selection.

Initial database searches identified 3555 articles. An additional five studies were identified via citation searching. After removal of duplicates, 1711 articles were retained for title and abstract screening. At this stage 1605 were removed leaving 106 articles for full text inspection. Screening of the full texts in relation to inclusion and exclusion criteria resulted in 47 studies to be included (see Data S1 for full reference list of included studies). The PRISMA flow diagram of study selection is presented in Figure 1 and details of included studies are presented in Table 1. Inter‐rater reliability for all stages was excellent (Cohen's kappa > 0.9).

**TABLE 1:** Included studies and exposures and outcomes investigated.

Study	Exposures investigated	Outcome	Method	Quality assessment
Abidin et al. (2021)	Age related hearing loss	AD	Two‐sample MR	Moderate
Anderson et al.(2020)	Educational attainment	AD	Two‐sample MR	High
Andrews et al. (2020)	Alcohol related exposures: alcohol consumption; alcohol dependence; AUDIT	AD; AAOS	Two‐sample MR	High
Andrews et al. (2021)	Alcohol related exposures: AUDIT, alcohol consumption BMI Depression Educational attainment Hearing difficulties Hypertension related exposures: diastolic blood pressure; systolic blood pressure Moderate to vigorous physical activity Smoking related exposures: cigarettes per day; smoking initiation Social isolation Type 2 diabetes	AD	Two‐sample MR	Moderate
Baumeister et al. (2020)	Physical activity related exposures: average physical activity; vigorous physical activity	AD	Two‐sample MR	High
Chen et al. (2022)	Education related exposures: college or university degree; O‐Levels/GCSEs or equivalents; no qualifications; age completed full time education Obesity related exposures: BMI; body fat percentage; whole body fat‐free mass Physical activity related exposure: time spent using a computer	AD	Two‐sample MR	High
Chen et al. (2023)	Obesity related exposures: BMI; waist‐hip ratio; waist‐hip ratio adjusted for BMI	AD & AD by proxy	Two‐sample MR	High
Desai et al. (2023)	Alcohol related exposures: alcohol consumption BMI Depression Educational attainment Hearing difficulties Hypertension related exposures: systolic blood pressure Moderate to vigorous physical activity Smoking related exposures: lifetime smoking duration, heaviness, and cessation Social isolation Type 2 diabetes	AD & AD by proxy, AD; DLB; FTD	Two‐sample MR	High
Garfield et al. (2021)	Diabetes related exposures: HbA1c; type 2 diabetes	AD	Two‐sample MR	High
Harerimana et al. (2022)	Depression	AD	Two‐sample MR	Low
He et al. (2022)	Physical activity related exposures: TV watching, computer use, driving	AD	Two‐sample MR	Moderate
Hu et al. (2024)	Depression	AD; VaD; PDD; DLB; FTD	Two‐sample MR	High
Huang et al. (2023)	Alcohol consumption Education level BMI Diabetes related exposures: Type 2 diabetes; fasting glucose; fasting insulin; 2 h glucose; HbA1c Hypertension related exposures: hypertension; systolic blood pressure; diastolic blood pressure Smoking related exposures: smoking initiation; cigarettes per day	AD	Two‐sample MR	Moderate
Korologou‐Linden et al. (2022)	Education related exposures: A‐Levels; college degree Obesity related exposure: whole body fat‐free mass Physical activity related exposure: self‐reported moderate physical activity	AD	Two‐sample MR	Moderate
Larsson et al. (2017)	Alcohol consumption Educational attainment Diabetes related exposures: type 2 diabetes; fasting glucose; fasting insulin Hypertension related exposures: diastolic blood pressure; systolic blood pressure Obesity related exposures: BMI; waist to hip ratio adjusted BMI Smoking related exposures: smoking quantity; smoking initiation; smoking cessation	AD	Two‐sample MR	Moderate
Li et al. (2021)	Obesity related exposures: BMI; waist to hip ratio; waist circumference; body fat percentage	AD	Two‐sample MR	Moderate
Liao et al. (2022)	Physical activity related exposures: fraction of acceleration > 425 m‐gravities; overall acceleration average; moderate to vigorous physical activity; vigorous physical activity	AD	Two‐sample MR	Low
Litkowski et al. (2023)	Diabetes related exposures: type 2 diabetes; HbA1c; fasting glucose; fasting insulin	AD; all‐cause dementia; VaD	One‐sample MR	Moderate
Liu et al. (2022)	Educational attainment	AD; AD by proxy; AD & AD by proxy	Two‐sample MR	Low
Luo et al. (2023)	Alcohol consumption Blood Education related exposures: number of years Hypertension related exposures: diastolic blood pressure; systolic blood pressure Smoking status Type 2 diabetes	AD	Two‐sample MR	High
Malik et al. (2021)	BMI HbA1c Overall physical activity Systolic blood pressure Smoking index	Incident dementia	Two‐sample MR	Moderate
Meng et al. (2022)	Diabetes related exposures: type 2 diabetes; fasting glucose; insulin levels	AD	Two‐sample MR	Low
Mitchell et al. (2020)	Hearing impairment	AD	Two‐sample MR	Low
Mukerjee et al. (2015)	BMI	AD; dementia	One‐sample MR	Moderate
Mulugeta et al. (2021)	Adiposity related exposures: unfavourable metabolic profile for BMI	AD	One‐sample MR Two‐sample MR	Moderate Low
Ning et al. (2023)	Air pollution related exposures: particulate matter 2.5 (PM_2.5_); particulate matter 10 (PM_10_); nitrogen dioxide (NO_2_); nitrogen oxide (NO_*X*_)	AD	Two‐sample MR	Low
Nordesthaard et al. (2022)	Smoking cumulative	All‐cause dementia; AD; non‐AD dementia	One‐sample MR Two‐sample MR	Moderate Moderate
Østergaard et al. (2015)	BMI Diabetes related exposures: fasting glucose; insulin resistance; type 2 diabetes Education related exposures: length of education, completing university Systolic blood pressure Smoking related exposures: smoking quantity; smoking initiation	Alzheimer's Disease	Two‐sample MR	Low
Ou et al. (2021)	Hypertension related exposures: diastolic blood pressure systolic blood pressure; pulse pressure	AD	Two‐sample MR	High
Pan et al. (2020)	Diabetes related exposures: HbA1c; type 2 diabetes; fasting glucose; fasting insulin; homeostasis model assessment–B‐cell function; homeostasis model assessment–insulin resistance	AD	Two‐sample MR	Low
Raghavan et al. (2019)	Educational attainment	AD	Two‐sample MR	Low
Shen et al. (2021)	Social isolation related exposures: regular pub attendance; regular gym attendance; regular religious group attendance; loneliness	AD; AD by proxy	Two‐sample MR	Moderate
Sproviero et al. (2021)	Hypertension related exposures: diastolic blood pressure; systolic blood pressure	AD	Two‐sample MR	Moderate
Thomassen et al. (2020)	Type 2 diabetes	AD	Two‐sample MR	Moderate
Thorp et al. (2022)	Alcohol related exposures: drinks per week BMI Depression Education related exposures: educational attainment; cognitive component of educational attainment; non‐cognitive component of educational attainment Hearing impairment Hypertension related exposures: diastolic blood pressure; systolic blood pressure Loneliness Physical activity Smoking related exposures: smoking initiation; cigarettes per day Type 2 diabetes	AD	Two‐sample MR	High
Walter et al. (2016)	Diabetes related exposures: type 2 diabetes; type 2 diabetes‐adiposity; type 2 diabetes–beta cell function; type 2 diabetes – insulin sensitivity; type 2 diabetes – other biological factors	AD	Two‐sample MR	Moderate
Wang et al. (2020)	Educational attainment	AD	Two‐sample MR	High
Wang et al. (2024)	BMI	AD	Two‐sample MR	High
Wu et al. (2021)	Physical activity	AD	Two‐sample MR	Moderate
Xue et al. (2023)	Type 2 diabetes	AD	Two‐sample MR	Moderate
Yang et al. (2021)	Physical activity related exposures: sedentary behaviour (TV watching); sedentary behaviour (computer use); sedentary behaviour (driving)	AD	Two‐sample MR	High
Zhang et al. (2020)	BMI Educational attainment Current smoking Diabetes related exposures: type 2 diabetes; fasting insulin; fasting glucose	AD	Two‐sample MR	Moderate
Zhang et al. (2022)	Physical activity related exposures: overall activity; sedentary behaviour; walking; moderate intensity activity	AD	Two‐sample MR	Moderate
Zhou et al. (2019)	Obesity related exposures: BMI; waist to hip ratio; waist circumference; waist to hip ratio adjusted BMI	AD	Two‐sample MR	High
Zhou et al. (2022)	Diabetes related exposures: fasting insulin; insulin resistance	AD	Two‐sample MR	Moderate
Zhu et al. (2023)	Smoking cigarettes per day	AD	Two‐sample MR	High
Zhuang et al. (2021)	Obesity	AD	Two‐sample MR	Low

*Note:* Please see Data S1 for the full reference list of included studies.

### 3.2 Description of Studies

The 47 studies meeting inclusion criteria represented 240 unique MR associations for 11 of the Lancet Commission modifiable dementia risk factors exposure categories and nine different dementia outcomes. There were no MR studies examining traumatic brain injury and dementia risk. See Tables 2 and 3 for a summary of risk factors and outcomes.

**TABLE 2:** Summary of included risk factor MR analyses (*n* = 240 association from 47 studies).

Risk factor category	Specific exposures	Number of analyses (%)
Early Life	Education‐related exposures Educational attainment College/university degree O‐levels or equivalent A‐levels or equivalent No qualifications Age completed education Length of education Cognitive component Non‐cognitive component	25 (10.4) 15 (6.3) 3 (1.3) 1 (0.4) 1 (0.4) 1 (0.4) 1 (0.4) 1 (0.4) 1 (0.4) 1 (0.4)
	Adiposity‐related exposures BMI Body fat percentage Whole body fat‐free mass Waist to hip ratio adj BMI Waist to hip ratio Waist circumference Unfavourable metabolic profile for BMI Obesity	34 (14.2) 18 (7.5) 2 (0.8) 2 (0.8) 5 (2.1) 2 (0.8) 2 (0.8) 2 (0.8) 1 (0.4)
Mid Life	Alcohol‐related exposures Alcohol consumption Alcohol dependence Alcohol use disorders identification test Drinks per week	15 (6.3) 10 (4.2) 2 (0.8) 2 (0.8) 1 (0.4)
	Blood pressure‐related exposures Diastolic blood pressure Systolic blood pressure Pulse pressure Hypertension	30 (12.5) 10 (4.2) 13 (5.4) 4 (1.7) 3 (1.6)
	Hearing loss‐related exposures Age‐related hearing loss Hearing difficulties Hearing impairment	7 (2.9) 4 (1.7) 1 (0.4) 2 (0.8)
	Air pollution‐related exposures Particulate matter 2.5 Particulate matter 10 Nitrogen dioxide Nitrogen oxides	4 (1.7) 1 (0.4) 1 (0.4) 1 (0.4) 1 (0.4)
	Depression	10 (4.2)
Late life	Diabetes‐related exposures Type 2 diabetes Type 2 diabetes adiposity Type 2 diabetes insulin sensitivity Type 2 diabetes other biological factors Type 2 diabetes β cell function HbAC1 2‐h postprandial glucose Fasting glucose Fasting insulin Homeostasis β cell function Homeostasis insulin resistance Insulin resistance Insulin levels	53 (22.1) 25 (10.4) 1 (0.4) 1 (0.4) 1 (0.4) 1 (0.4) 6 (2.5) 3 (1.3) 5 (2.1) 5 (2.1) 1 (0.4) 1 (0.4) 2 (0.8) 1 (0.4)
	Physical activity‐related exposures Overall physical activity Vigorous physical activity Moderate to vigorous physical activity Moderate physical activity Walking Sedentary behaviour Sedentary behaviour: computer use Sedentary behaviour: TV watching Sedentary behaviour: driving Fractions of accelerations > 425 m‐gravities Overall acceleration	22 (9.2) 7 (2.9) 5 (2.1) 1 (0.4) 1 (0.4) 1 (0.4) 1 (0.4) 2 (0.8) 1 (0.4) 1 (0.4) 1 (0.4) 1 (0.4)
	Smoking‐related exposures Current smoking Lifetime smoking Smoking initiation Smoking quantity Smoking cessation Smoking cumulative Smoking index Smoking cigarettes per day Smoking regularly	25 (10.4) 1 (0.4) 2 (0.8) 6 (2.5) 2 (0.8) 1 (0.4) 4 (1.7) 1 (0.4) 6 (2.5) 2 (0.8)
	Social contact‐related exposures Social isolation Regular social club or pub attendance Regular sport club or gym attendance Regular religious group attendance Loneliness	15 (6.3) 1 (0.4) 2 (0.8) 2 (0.8) 2 (0.8) 8 (3.3)

Abbreviation: BMI, body mass index.

**TABLE 3:** Summary of dementia outcomes.

Dementia outcome	Number of analyses (%)
Alzheimer's disease (AD)	149 (62.1%)
AD by proxy	14 (5.8%)
Maternal AD	14 (5.8%)
Paternal AD	14 (5.8%)
Combined AD and AD by proxy	8 (3.3%)
AD age of onset	3 (1.3%)
All‐cause dementia	11 (4.6%)
Non‐AD dementia	1 (0.4%)
Dementia with Lewy bodies (DLB)	10 (4.2%)
Frontotemporal dementia (FTD)	11 (4.6%)
Parkinson's disease dementia (PDD)	1 (0.4%)
Vascular dementia (VaD)	4 (1.7%)

### 3.3 Quality Assessment

18 (38.3%) studies were rated ‘high’ quality, 19 (40.4%) studies were rated ‘moderate’ quality and 11 studies (23.4%) were rated ‘low’ quality. The 11 studies given a ‘low’ quality rating were removed before the evidence was evaluated.

### 3.4 Evaluation of Evidence

**FIGURE 2:**
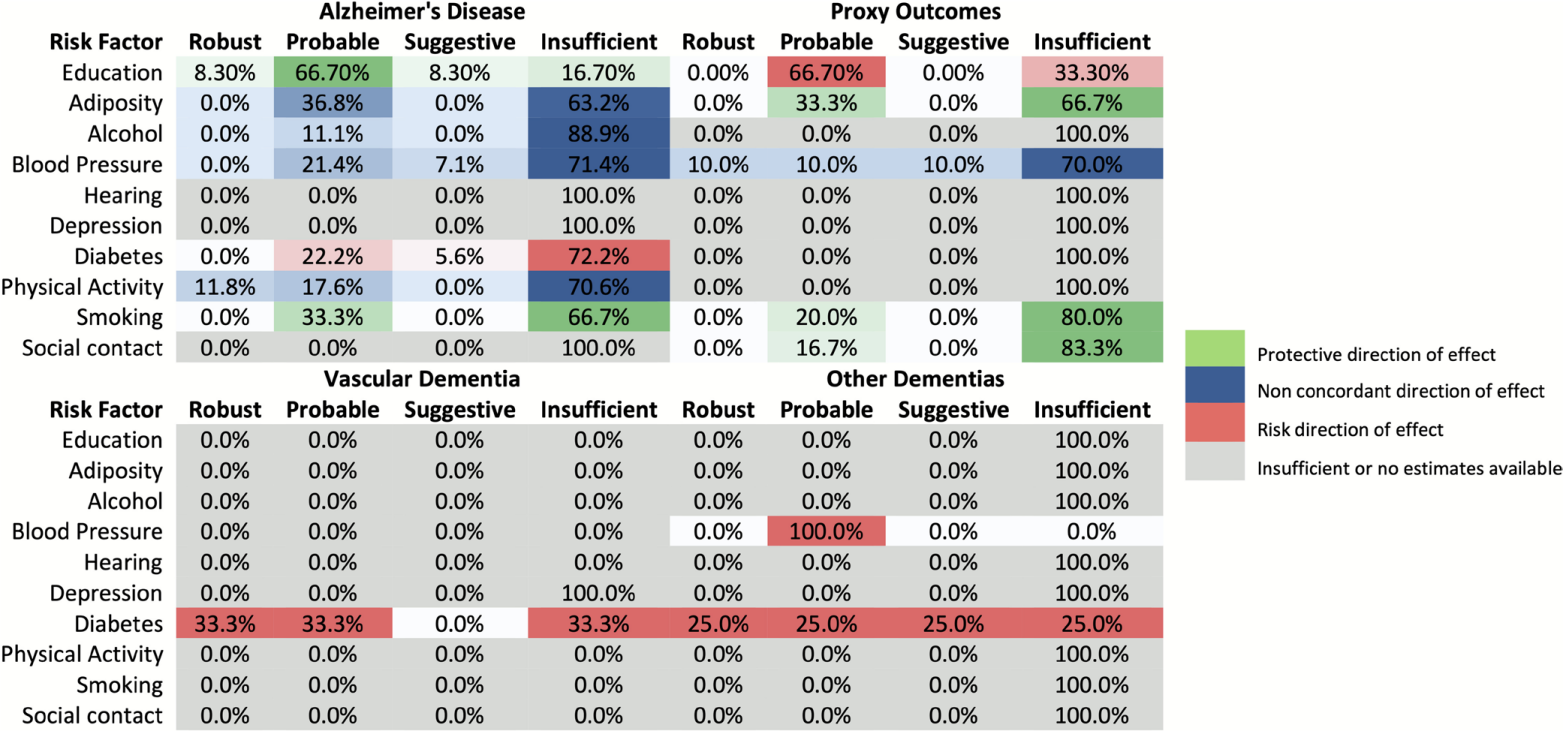
Proportion of evidence strength by outcome for Alzheimer's disease, proxy diagnosed outcomes, vascular dementia and other dementias.

The proportion of estimates in each category is displayed in Figure 2. For all the exposures aside from education‐related and depression‐related exposures, the bulk (> 50%) of evidence was graded as providing ‘insufficient’ evidence of causal links.

#### 3.4.1 Clinically Diagnosed Outcomes

For AD evaluable analyses were available for all risk factors. Among early‐life exposures, education‐related factors showed mostly probable evidence (66.7%, 8/12) for a protective association, with a smaller proportion of analyses graded as robust (8.3%) or suggestive (8.3%). In contrast, mid‐life exposures such as adiposity (*n* = 19), alcohol consumption (*n* = 9), and blood pressure (*n* = 14) were predominantly graded as providing insufficient evidence (> 60% in each case). For adiposity, most analyses focused on body mass index (BMI) (*n* = 12), of which three were graded as probable but showed inconsistent directions of effect. One analysis examined waist‐to‐hip ratio and indicated a probable risk association, while three probable analyses of body fat measures also showed inconsistent directions of effect. Although 21.4% of blood pressure analyses were graded as probable and 7.1% as suggestive, there was no concordant direction of effect across estimates. All analyses for hearing loss (*n* = 3) were graded insufficient.

Among late‐life exposures for AD, there was no robust or probable evidence for depression (*n* = 2) or social contact (*n* = 7); all analyses were graded as insufficient. For diabetes‐related exposures (*n* = 19), 22.2% were graded as probable and 5.6% as suggestive, with the remainder insufficient; all concordant effects pointed in the direction of increased risk. Physical activity (*n* = 17) showed some robust (11.8%) and probable (17.6%) evidence, but the majority (70.6%) of analyses were insufficient. For smoking (*n* = 9), 33.3% were graded as probable with the direction of effect consistently suggesting a protective association.

For vascular dementia (VaD), three evaluable analyses were identified (for diabetes), which provided probable evidence of increased risk. For dementia with Lewy bodies (DLB) and frontotemporal dementia (FTD), evaluable MR analyses were limited (typically 1 per risk factor) and all were graded as providing insufficient evidence. For all‐cause or other forms of dementia, most risk factors again yielded insufficient evidence. A notable exception was for diabetes, where four analyses (one each graded as robust, probable, suggestive, and insufficient) were available, with the direction of effect consistently aligned with increased dementia risk. See Supplementary Table 2 for the number and percentage of ‘high’ or ‘moderate’ quality studies per category by modifiable risk factor.

#### 3.4.2 Proxy Outcomes

All proxy outcomes assessed familial history of AD. When restricting to analyses using proxy‐diagnosed dementia, evidence was generally weaker. For AD proxy outcomes, education (*n* = 3) showed two graded as probable and one insufficient, with there being a risk direction of effect. Adiposity‐related exposures (*n* = 3) yielded one probable analysis in the protective direction. Blood pressure (*n* = 10) was the only risk factor with analyses graded across all four evidence categories, including one robust analysis, although the direction of effect was not concordant. All proxy analyses for hearing loss (*n* = 1), depression (*n* = 1), diabetes (*n* = 12), and physical activity (*n* = 1) were graded as insufficient. For smoking (*n* = 5), one analysis (20%) showed probable evidence suggesting a protective association, while the rest were insufficient. Similarly, one of six analyses for social contact (16.7%) was graded as probable, with the remainder insufficient, and the direction of effect suggested a protective relationship. See Supplementary Table 3 for the number and percentage of ‘high’ or ‘moderate’ quality studies per category by modifiable risk factor.

For all estimates given a ‘robust’, ‘probable’ or ‘suggestive’ grading we further examined the direction of effect across the risk factors stratified by either clinically diagnosed dementia or proxy outcomes. For clinically diagnosed outcomes, all the educational attainment and diabetes‐related exposures were in the expected direction. However, smoking appeared to have a protective association with dementia. Adiposity, alcohol consumption, blood pressure and physical activity had non‐concordant directions of effect across the studies. For the proxy outcomes, there was a similar pattern of results with a notable exception of educational attainment which had one estimate pointing in the risk direction.

### 3.5 Post‐Hoc Sensitivity Analysis

During data extraction and quality appraisal, we identified several GWAS exposure datasets where concerns were raised about construct validity, i.e., whether the genetic instrument accurately captured the intended risk factor. To examine the impact of these potential biases, we conducted a post hoc sensitivity analysis by removing these exposures from the synthesis. To ensure greater methodological rigor, only clinically diagnosed dementia outcomes were retained in this analysis, allowing us to focus on estimates with the most valid exposure and outcome measures. Exposures flagged for removal included measures unlikely to validly capture the intended risk factor, such as “computer use” and “driving” as proxies for physical activity, and “gym attendance” and “pub visits” as indicators of social contact. In total, 11 estimates were excluded across three risk factor categories (adiposity, physical activity, and social contact). Removing one adiposity‐related estimate measuring an unfavorable metabolic profile for BMI had no impact on the synthesis, as the estimate was non‐evaluable. For physical activity, four estimates were removed, two graded as ‘insufficient’ and two as either ‘robust’ or ‘probable’ and in the risk direction. Following their exclusion, the remaining evidence included three ‘probable’ estimates (two in the protective direction, one in the risk direction) and one ‘robust’ estimate (in the protective direction), suggesting increased consistency in the direction of effect. For social contact, six insufficient estimates based on regular gym, pub, or religious group attendance were excluded; the one ‘probable’ estimate removed in this group was based on a proxy outcome and therefore not relevant to this analysis. Overall, the sensitivity analysis did not materially alter the results.

## 4 Discussion

We aimed to summarise the MR evidence for causal relationships between 12 modifiable risk factors and dementia outcomes. Across 104 unique and evaluable MR analyses, the majority of estimates were graded as providing insufficient evidence for a causal association between genetically predicted exposures and dementia outcomes. For clinically diagnosed Alzheimer's disease, the strongest evidence was observed for education and diabetes‐related exposures, with most estimates for education graded as probable and pointing toward a protective association, and diabetes estimates indicating increased dementia risk with high concordance in direction. In contrast, mid‐life exposures such as adiposity, alcohol use, and blood pressure produced mixed or inconclusive results, with substantial heterogeneity in effect direction. Smoking‐related exposures showed a consistent protective direction of effect. For other risk factors, including hearing loss, depression, and social contact, the available MR evidence was consistently insufficient. Results for other dementia subtypes and proxy outcomes were generally limited or weaker in quality. A post hoc sensitivity analysis, which excluded estimates based on exposures with low construct validity, did not materially change the pattern of findings but improved internal consistency for physical activity and social contact categories.

When stratified by risk factor and outcome, several important patterns emerged. Among early‐life exposures, education consistently demonstrated probable or robust evidence for a protective association with clinically diagnosed AD. All the evidence for this risk factor and other dementia subtypes was classified as ‘insufficient’. However, this association reversed direction when proxy outcomes were used, highlighting concerns about measurement validity in GWAS relying on family history. This discrepancy highlights the growing body of evidence showing that GWAS‐by‐proxy designs, particularly for AD, are vulnerable to participation, misclassification, and survival biases that may undermine downstream MR analyses [8]. However, in line with the findings of Wu et al. [8], our analyses indicated that the use of proxy outcomes had the most pronounced impact on the exposure of educational attainment, with estimates reversing direction compared to those based on clinically diagnosed outcomes. For the remaining risk factors, eliminating proxy outcomes had little impact on the overall pattern of results, suggesting that these estimates were either consistently null or less sensitive to the biases introduced by proxy phenotyping.

For mid‐life exposures, the findings were more mixed. Adiposity‐related traits showed probable evidence of association with AD, although directionality was inconsistent across the studies. Measures of waist‐to‐hip ratio and body fat mass showed both risk and protective associations depending on the outcome and dataset. Alcohol‐related exposures yielded mostly insufficient evidence for all dementia outcomes. For blood pressure, analyses of systolic blood pressure (SBP), diastolic blood pressure (DBP), pulse pressure (PP), and hypertension status did not converge on a consistent direction of effect across dementia outcomes. Notably, while the analyses for SBP and DBP showed probable associations with AD two out of three were in the protective direction of effect. This may reflect survivor bias or suggest that AD is less influenced by elevated blood pressure than other dementia subtypes such as vascular dementia, for which evaluable estimates and associations with blood pressure traits were unavailable. It is important to note that no estimates were available for blood pressure traits and the risk of VaD.

There was some evidence that smoking may have a mitigating effect on AD risk. However, this result needs to be interpreted with caution because there is strong evidence to link smoking to overall negative health outcomes and premature all‐cause mortality [10]. In addition, the results that are observed in MR studies may be an artefact of survivor bias [11]. Individuals who smoke may die earlier of other smoking‐related diseases and therefore may not survive long enough to develop an age‐related disease. As such what appears to be a link with dementia may be an association with longevity.

One risk factor namely type 2 diabetes and related biomarkers had the most evidence of causal links to AD, VaD and all‐cause dementia outcomes with the direction of effect aligned across analyses in the risk direction. This was the risk factor that had comparatively the strongest overall evidence to be causally linked to dementia.

### 4.1 Limitations and Future Research

MR studies for dementia face several challenges, notably in identifying valid instrumental variables, construct validity, survivor bias and lack of subtype‐specific analyses. For IVs to be valid there must be a biologically plausible pathway between the IV exposure and outcome. The most notable case of this limitation is that of the one study investigating the effect of air pollution on dementia [12]. These analyses were all deemed non‐evaluable due to concerns over the lack of biological plausibility of the IV [13]. Studies may be limited in how well the exposure or outcome in question is operationalized. Exposures such as physical activity and social contact were susceptible to construct validity bias. Physical inactivity in one study [14] was assessed as time spent on a computer when this may also be a measure of mental activity or work. Likewise, in another study social contact was operationalized as regular gym attendance [15] when this could also be a measure of physical activity rather than social contact. Reliance on proxy phenotypes can lead to misclassification and bias the accuracy and reliability of study findings. However, our study stratified for proxy outcomes and conducted sensitivity analyses to assess the impact of construct validity and the pattern of results was largely unchanged. Survivor bias complicates all analyses of age‐dependent outcomes, including MR studies, observational studies, and randomized controlled trials. However, in MR specifically, this can present challenges in later‐life outcomes like dementia, where individuals with certain genetic predispositions may not survive to the age of outcome assessment. There is emerging evidence that different subtypes of dementia may be differentially impacted by some risk factors; specifically Desai et al. [16] reported that hypertension may have a greater impact on the risk of VaD than on AD. However, the availability of detailed data on dementia subtypes, particularly VaD, DLB and FTD is limited.

Most of the included MR studies used GWAS summary data derived from populations of predominantly European ancestry, often sourced from large‐scale biobanks such as UK Biobank. While these resources offer substantial statistical power and broad phenotypic coverage, they also introduce limitations in generalisability to non‐European populations. Additionally, several dementia GWAS used in the included MR studies [17] pooled data across studies with varying recruitment criteria and did not always ensure rigorous age‐matching between cases and controls. The use of biobank‐derived exposures may also reflect relatively healthy, educated populations and could contribute to healthy volunteer bias. These issues should be considered when interpreting the strength and scope of evidence across different risk factors and dementia subtypes, and highlight the need for future MR studies using ancestrally diverse, age‐matched, and clinically validated samples. Another important limitation of this review is that here we only consider the risk factors for dementia as identified by the Lancet Commission 2020. MR studies in the field of dementia research have focused on a broad range of modifiable risk factors such as coffee consumption [18] as well as many biomarkers [19]. Future reviews should focus on evaluating the evidence from non‐Lancet Commission risk modifiable factors as well as those additional risk factors identified in the most recent iteration of the Lancet Commission [20].

## 5 Conclusion

Currently, most of the evidence base for causal relationships between ten of the Lancet commission risk factors for dementia is insufficient when the evidence from MR studies is considered. However, educational attainment and type 2 diabetes‐related dysfunction were the two risk factors that had the most evidence of causal links to AD, for education, and all dementia subtypes for diabetes. The null results for other exposures may reflect limitations such as low statistical power, particularly when genetic instruments explain a small proportion of trait variance, or violations of MR assumptions.

## Author Contributions

**Roopal Desai:** conceptualisation, formal analyses, investigation, methodology, writing – original draft. **Amber John:** formal analyses, validation, writing – review and editing. **Emma Anderson:** formal analyses, validation, writing – review and editing. **Jean Stafford:** formal analyses, validation, writing – review and editing. **Aysha Mohamed Rafik Patel:** writing review and editing. **Natalie L. Marchant:** writing – review and editing. **Georgina Charlesworth:** writing – review and editing. **Verena Zuber:** writing – review and editing. **Joshua Stott:** supervision, writing – review and editing.

## Funding

The authors have nothing to report.

## Conflicts of Interest

The authors declare no conflicts of interest.

## Supporting information


**Table S1:** Results of included studies investigating modifiable risk factors and dementia.
**Table S2:** Number and percentage of ‘high’ or ‘moderate’ quality, unique Mendelian randomisation analyses per category by modifiable risk factor for clinically diagnosed dementia outcomes.
**Table S3:** Number and percentage of ‘high’ or ‘moderate’ quality, unique Mendelian randomisation analyses per category by modifiable risk factor for proxy outcomes.

## Data Availability

Data sharing not applicable to this article as no datasets were generated or analysed during the current study.
