# Supplementary material for: Evidence for Causal Links Between Known Modifiable Risk Factors and Dementia: A Systematic Review of Mendelian Randomisation Studies
Source: Eur J Neurol. 2025 Dec 4;32(12):e70458. doi: 10.1111/ene.70458 (PMC12676184; doi:10.1111/ene.70458)
Supplement: Supplementary file 1 — Table S1: Results of included studies investigating modifiable risk factors and dementia. Table S2: Number and percentage of ‘high’ or ‘moderate’ quality, unique Mendelian randomisation analyses per category by modifiable risk factor for clinically diagnosed dementia outcomes. Table S3: Number and percentage of ‘high’ or ‘moderate’ quality, unique Mendelian randomisation analyses per category by modifiable risk factor for proxy outcomes. [file ENE-32-e70458-s001.docx]

Supplementary Table1: Results of included studies investigating modifiable risk factors and dementia

| **Modifiable Risk Factor** | **Study** | **Exposure (GWAS dataset/consortium)** | **Outcome (GWAS dataset/consortium)** | **N SNPs** | ***F*-statistic** | **IVW Estimate** |
| --- | --- | --- | --- | --- | --- | --- |
| **Early Life** |  |  |  |  |  |  |
| **Education related exposures** | Anderson et al.,(2020)^2^ | Educational attainment (Okbay et al., 2016^48^) | Alzheimer’s Disease (Lambert et al., 2013^49^) | 142 | 43.5 | OR=0.62 95% CI:0.51;0.77* |
|  | Andrews et al., (2021)^4^ | Educational attainment (Lee et al., 2018^50^) | Alzheimer’s Disease (Kunkle et al., 2019^51^) | 478 | 55.9 | β=-0.44 SE=0.07* |
|  | Chen et al., (2022)^6^ | College or University degree (MRC-IEU consortium) | Alzheimer’s Disease (Kunkle et al., 2019^51^) | 211 | 46.7 | OR=0.47 95% CI:0.34;0.67* |
|  |  | O-Levels or equivalent (MRC-IEU consortium) | Alzheimer’s Disease (Kunkle et al., 2019^51^) | 18 | 35.6 | OR=0.28 95% CI:0.09;0.89 |
|  |  | No qualifications (MRC-IEU consortium) | Alzheimer’s Disease (Kunkle et al., 2019^51^) | 78 | 42.1 | OR=4.01 95% CI:1.95;8.21 |
|  |  | Age completed education (MRC-IEU consortium) | Alzheimer’s Disease (Kunkle et al., 2019^51^) | 37 | 36 | OR=0.76 95% CI:0.48;1.20 |
|  | Desai et al., (2023)^8^ | Educational attainment (Okbay et al., 2016^48^) | Alzheimer’s Disease and Alzheimer’s Disease by Proxy (Bellenguez et al., 2023^52^) | 70 | 45.8 | OR=1.93 95% CI:1.05;3.54 |
|  |  | Educational attainment (Okbay et al., 2016^48^) | Dementia with Lewy Bodies (Chia et al., 2021^53^) | 67 | 45.5 | OR=1.99 95% CI:0.24;16.23 |
|  |  | Educational attainment (Okbay et al., 2016^48^) | Frontotemporal Dementia (Ferrari et al., 2014^54^) | 71 | 46 | OR=2.84 95% CI:0.24;33.57 |
|  | Huang et al., (2023)^13^ | Educational attainment (Lee et al., 2018^50^) | Alzheimer’s Disease (Kunkle et al., 2019^51^) | 262 | NR | OR=0.70 95% CI:0.60;0.81* |
|  |  | Educational attainment (Lee et al., 2018^50^) | Alzheimer’s Disease by Proxy -Maternal history (Marioni et al., 2018^55^) | 262 | NR | OR=1.31 95% CI:1.10;1.57 |
|  |  | Educational attainment (Lee et al., 2018^50^) | Alzheimer’s Disease by Proxy -Paternal history (Marioni et al., 2018^55^) | 262 | NR | OR=1.21 95% CI:0.94;1.56 |
|  | Korologou-Linden et al., (2022)^14^ | A-Levels (UK Biobank) | Alzheimer’s Disease (Lambert et al., 2013^49^) | 77 | >30 | OR=0.82 95% CI:0.73;0.91* |
|  |  | College degree (UK Biobank) | Alzheimer’s Disease (Lambert et al., 2013^49^) | 241 | >30 | OR=0.82 95% CI:0.76;0.88* |
|  | Larsson et al., (2017)^15^ | Educational attainment (Okbay et al., 2016^48^) | Alzheimer’s Disease (Lambert et al., 2013^49^) | 152 | 5.7 | OR=0.89 95% CI:0.84;0.93* |
|  | Liu et al., (2022)^19^ | Educational attainment (Okbay et al., 2016^48^) | Alzheimer’s Disease (Kunkle et al., 2019^51^) | 159 | NR | OR=0.68 95% CI:0.57;0.81* |
|  |  | Educational attainment (Okbay et al., 2016^48^) | Alzheimer’s Disease by proxy (Marioni et al., 2018^55^) | 147 | NR | OR=1.09 95% CI:1.00;1.19 |
|  |  | Educational attainment (Okbay et al., 2016^48^) | Alzheimer’s Disease by proxy (Schwartzentruber et al., 2021^56^) | 159 | NR | OR=1.88 95% CI:1.62;2.18* |
|  |  | Educational attainment (Okbay et al., 2016^48^) | Alzheimer’s Disease & Alzheimer’s Disease by proxy (Marioni et al., 2018^55^) | 147 | NR | OR=0.99 95% CI:0.91;1.07 |
|  |  | Educational attainment (Okbay et al., 2016^48^) | Alzheimer’s Disease & Alzheimer’s Disease by proxy (Jansen et al., 2019^57^) | 155 | NR | OR=0.96 95% CI:0.93;0.98* |
|  |  | Educational attainment (Okbay et al., 2016^48^) | Alzheimer’s Disease & Alzheimer’s Disease by proxy (Schwartzentruber et al., 2021^56^) | 159 | NR | OR=1.21 95% CI:1.08;1.36* |
|  | Luo et al., (2023)^20^ | Educational attainment (Okbay et al., 2016^48^) | Alzheimer’s Disease (Bellenguez et al., 2022^52^) | 3201 | >10 | OR=0.83 95% CI:0.79;0.87* |
|  |  | Educational attainment (Okbay et al., 2016^48^) | Alzheimer’s Disease by proxy (Bellenguez et al., 2022^52^) | 3197 | >10 | OR=1.06 95% CI:1.01;1.10 |
|  | Østergaard et al., (2015)^28^ | Length of education (Rietveld et al., 2013^58^) | Alzheimer’s Disease (Lambert et al., 2013^49^) | 1 | NR | OR=0.71 95% CI:0.48;1.06 |
|  |  | Completing university (Rietveld et al., 2013^58^) | Alzheimer’s Disease (Lambert et al., 2013^49^) | 2 | NR | OR=0.95 95% CI:0.67;1.34 |
|  | Raghavan et al., (2019)^31^ | Educational attainment (Okbay et al., 2016^48^) | Alzheimer’s Disease (Lambert et al., 2013^49^) | 1271 | NR | OR=0.63 95% CI:0.54;0.74* |
|  | Thorp et al., (2022)^35^ | Educational attainment (Lee et al., 2018^50^) | Alzheimer’s Disease (Lambert et al., 2013^49^) | 271 | 48.8 | OR=0.66 95% CI:0.55;0.78* |
|  |  | Cognitive component of educational attainment (Lee et al., 2018^50^ and Savage et al., 2018^59^) | Alzheimer’s Disease (Lambert et al., 2013^49^) | 121 | 42.4 | OR=0.85 95% CI:0.78;0.92* |
|  |  | Non cognitive component of educational attainment (Lee et al., 2018^50^ and Savage et al., 2018^59^) | Alzheimer’s Disease (Lambert et al., 2013^49^) | 28 | 36.9 | OR=0.94 95% CI:0.82;1.08 |
|  | Wang et al., (2020)^37^ | Educational attainment (Okbay et al., 2016^48^) | Alzheimer’s Disease (Lambert et al., 2013^49^) | 64 | NR | OR=0.59 95% CI:0.45;0.77* |
|  | Zhang et al., (2020)^42^ | Educational attainment (Lee et al., 2018^50^) | Alzheimer’s Disease (Lambert et al., 2013^49^) | 248 | NR | OR=0.67 95% CI:0.57;0.80* |
| **Mid-Life** |  |  |  |  |  |  |
| **Alcohol consumption related exposures** | Andrews et al., (2020)^3^ | Alcohol consumption (Liu et al., 2019^60^) | Alzheimer’s Disease (Lambert et al., 2013^49^) | 44 | 70.8 | OR=0.96 95% CI:0.74;1.25 |
|  |  | Alcohol dependence (Walter et al., 2018^61^) | Alzheimer’s Disease (Lambert et al., 2013^49^) | 20 | 24.6 | OR=0.93 95% CI:0.93;1.04 |
|  |  | AUDIT (Sanchez-Roige et al., 2019^62^) | Alzheimer’s Disease (Lambert et al., 2013^49^) | 11 | 59.3 | OR=0.45 95% CI:0.12;1.75 |
|  |  | Alcohol consumption (Liu et al., 2019^60^) | Alzheimer’s Disease Age of Onset Survival (Huang et al., 2017^63^) | 44 | 70.8 | HR=2.02 95% CI:1.42;2.87* |
|  |  | Alcohol dependence (Walter et al., 2018^61^) | Alzheimer’s Disease Age of Onset Survival (Huang et al., 2017^63^) | 20 | 24.6 | HR=0.93 95% CI:0.87;0.99 |
|  |  | AUDIT (Sanchez-Roige et al., 2019^62^) | Alzheimer’s Disease Age of Onset Survival (Huang et al., 2017^63^) | 11 | 59.3 | β=0.26 SE=0.18 |
|  | Andrews et al., (2021)^4^ | AUDIT (Sanchez-Roige et al., 2019^62^) | Alzheimer’s Disease (Kunkle et al., 2019^51^) | 12 | 58.2 | β=0.59 SE=0.29 |
|  |  | Alcohol consumption (Liu et al., 2019^60^) | Alzheimer’s Disease (Kunkle et al., 2019^51^) | 86 | 69.4 | β=0.28 SE=0.26 |
|  | Desai et al., (2023)^8^ | Alcohol consumption (Evangelou et al., 2019)^64^ | Alzheimer’s Disease and Alzheimer’s Disease by Proxy (Bellenguez et al., 2023^52^) | 72 | 45.8 | OR=1.19 95% CI:0.85;1.66 |
|  |  | Alcohol consumption (Evangelou et al., 2019)^64^ | Dementia with Lewy Bodies (Chia et al., 2021^53^) | 69 | 58 | OR=0.60 95% CI:0.18;2.03 |
|  |  | Alcohol consumption (Evangelou et al., 2019)^64^ | Frontotemporal Dementia (Ferrari et al., 2014^54^) | 65 | 61.9 | OR=1.54 95% CI:1.20;1.97 |
|  | Huang et al., (2023)^13^ | Alcohol consumption (Liu et al., 2019^60^) | Alzheimer’s Disease (Kunkle et al., 2019^51^) | 32 | NR | OR=1.22 95% CI:0.83;1.78 |
|  |  | Alcohol consumption (Liu et al., 2019^60^) | Alzheimer’s Disease by Proxy -Maternal history (Marioni et al., 2018^55^) | 32 | NR | OR=1.27 95% CI:0.84;1.94 |
|  |  | Alcohol consumption (Liu et al., 2019^60^) | Alzheimer’s Disease by Proxy -Paternal history (Marioni et al., 2018^55^) | 32 | NR | OR=0.95 95% CI:0.53;1.69 |
|  | Larsson et al., (2017)^15^ | Alcohol consumption (Jorgenson et al., 2017^65^) | Alzheimer’s Disease (Lambert et al., 2013^49^) | 3 | NR | OR=0.72 95% CI:0.5;1.04 |
|  | Luo et al., (2023)^20^ | Alcohol consumption (Liu et al., 2019^60^) | Alzheimer’s Disease (Bellenguez et al., 2022^52^) | 77 | >10 | OR=0.87 95% CI:0.70;1.07 |
|  |  | Alcohol consumption (Liu et al., 2019^60^) | Alzheimer’s Disease by proxy (Bellenguez et al., 2022^52^) | 78 | >10 | OR=0.93 95% CI:0.74;1.15 |
|  | Thorp et al., (2022)^35^ | Drinks per week (Liu et al., 2019^60^) | Alzheimer’s Disease (Lambert et al., 2013^49^) | 29 | 76.4 | OR=1.08 95% CI:0.71;1.65 |
| **Blood pressure related exposures** | Andrews et al., (2021)^4^ | Diastolic blood pressure (Evangelou et al., 2018^66^) | Alzheimer’s Disease (Kunkle et al., 2019^51^) | 450 | 82.6 | β=-0.001 SE=0.004 |
|  |  | Systolic blood pressure (Evangelou et al., 2018^66^) | Alzheimer’s Disease (Kunkle et al., 2019^51^) | 435 | 78.1 | β=-0.005 SE=0.003 |
|  | Desai et al., (2023)^8^ | Systolic blood pressure (UKBiobank) | Alzheimer’s Disease and Alzheimer’s Disease by Proxy (Bellenguez et al., 2023^52^) | 219 | 45.4 | OR=0.90 95% CI:0.82;0.99* |
|  |  | Systolic blood pressure (UKBiobank) | Dementia with Lewy Bodies (Chia et al., 2021^53^) | 213 | 45.6 | OR=1.12 95% CI:0.82;1.52 |
|  |  | Systolic blood pressure (UKBiobank) | Frontotemporal Dementia (Ferrari et al., 2014^54^) | 212 | 45.1 | OR=0.95 95% CI:0.67;1.37 |
|  | Huang et al., (2023)^13^ | Hypertension (UK Biobank) | Alzheimer’s Disease (Kunkle et al., 2019^51^) | 143 | NR | OR=1.45 95% CI:1.03;2.03 |
|  |  | Hypertension (UK Biobank) | Alzheimer’s Disease by Proxy -Maternal history (Marioni et al., 2018^55^) | 138 | NR | OR=1.50 95% CI:0.98;2.30 |
|  |  | Hypertension (UK Biobank) | Alzheimer’s Disease by Proxy -Paternal history (Marioni et al., 2018^55^) | 138 | NR | OR=2.24 95% CI:1.26;4.00 |
|  |  | Diastolic blood pressure (UK Biobank) | Alzheimer’s Disease (Kunkle et al., 2019^51^) | 398 | NR | OR=0.95 95% CI:0.67;1.37 |
|  |  | Diastolic blood pressure (UK Biobank) | Alzheimer’s Disease by Proxy -Maternal history (Marioni et al., 2018^55^) | 374 | NR | OR=1.00 95% CI:0.98;1.01 |
|  |  | Diastolic blood pressure (UK Biobank) | Alzheimer’s Disease by Proxy -Paternal history (Marioni et al., 2018^55^) | 374 | NR | OR=0.98 95% CI:0.96;0.99 |
|  |  | Systolic blood pressure (UK Biobank) | Alzheimer’s Disease (Kunkle et al., 2019^51^) | 400 | NR | OR=1.00 95% CI:0.99;1.00 |
|  |  | Systolic blood pressure (UK Biobank) | Alzheimer’s Disease by Proxy -Maternal history (Marioni et al., 2018^55^) | 380 | NR | OR=0.99 95% CI:0.98;1.00 |
|  |  | Systolic blood pressure (UK Biobank) | Alzheimer’s Disease by Proxy -Paternal history (Marioni et al., 2018^55^) | 380 | NR | OR=0.99 95% CI:0.98;1.00 |
|  |  | Pulse Pressure (UK Biobank) | Alzheimer’s Disease (Kunkle et al., 2019^51^) | 343 | NR | OR=1.00 95% CI:0.98;1.01 |
|  |  | Pulse Pressure (UK Biobank) | Alzheimer’s Disease by Proxy -Maternal history (Marioni et al., 2018^55^) | 326 | NR | OR=0.99 95% CI:0.98;1.01 |
|  |  | Pulse Pressure (UK Biobank) | Alzheimer’s Disease by Proxy -Paternal history (Marioni et al., 2018^55^) | 326 | NR | OR=0.99 95% CI:0.98;1.01 |
|  | Larsson et al., (2017)^15^ | Diastolic blood pressure (Hoffmann et al., 2017^67^) | Alzheimer’s Disease (Lambert et al., 2013^49^) | 105 | 11 | OR=0.96 95% CI:0.79;1.16 |
|  |  | Systolic blood pressure (Hoffmann et al., 2017^67^) | Alzheimer’s Disease (Lambert et al., 2013^49^) | 93 | 15 | OR=0.94 95% CI:0.77;1.14 |
|  | Luo et al., (2023)^20^ | Diastolic blood pressure (Evangelou et al., 2018^66^) | Alzheimer’s Disease (Bellenguez et al., 2022^52^) | 216 | >10 | OR=1.02 95% CI:0.96;1.09 |
|  |  | Diastolic blood pressure (Evangelou et al., 2018^66^) | Alzheimer’s Disease by proxy (Bellenguez et al., 2022^52^) | 215 | >10 | OR=0.98 95% CI:0.93;1.03 |
|  |  | Systolic blood pressure (Evangelou et al., 2018^66^) | Alzheimer’s Disease (Bellenguez et al., 2022^52^) | 268 | >10 | OR=0.89 95% CI:0.81;0.98* |
|  |  | Systolic blood pressure (Evangelou et al., 2018^66^) | Alzheimer’s Disease by proxy (Bellenguez et al., 2022^52^) | 268 | >10 | OR=0.85 95% CI:0.78;0.92* |
|  | Malik et al., (2021)^21^ | Systolic blood pressure (UKBiobank) | Incident Dementia (UK Biobank) | 460 | NR | OR=1.31 95% CI:1.05;1.60 |
|  | Østergaard et al., (2015)^28^ | Systolic blood pressure (Ehret et al., 2011^68^) | Alzheimer’s Disease (Lambert et al., 2013^49^) | 24 | NR | OR=0.75 95% CI:0.62;0.91 |
|  | Ou et al., (2021)^29^ | Diastolic blood pressure (Evangelou et al., 2018^66^) | Alzheimer’s Disease (Kunkle et al., 2019^51^) | 398 | NR | OR=0.99 95% CI:0.98;1.00 |
|  |  | Systolic blood pressure (Evangelou et al., 2018^66^) | Alzheimer’s Disease (Kunkle et al., 2019^51^) | 400 | NR | OR=1.00 95% CI:0.99;1.01 |
|  |  | Pulse pressure (Evangelou et al., 2018^66^) | Alzheimer’s Disease (Kunkle et al., 2019^51^) | 343 | NR | OR=1.00 95% CI:0.98;1.01 |
|  | Sproviero et al., (2021)^33^ | Diastolic blood pressure (Evangelou et al., 2018^66^) | Alzheimer’s Disease (Lambert et al., 2013^49^) | 56 | NR | OR=0.95 95% CI:0.80;1.10 |
|  |  | Systolic blood pressure (Evangelou et al., 2018^66^) | Alzheimer’s Disease (Lambert et al., 2013^49^) | 63 | NR | OR=0.91 95% CI:0.83;0.99 |
|  | Thorp et al., (2022)^35^ | Diastolic blood pressure (Evangelou et al., 2018^66^) | Alzheimer’s Disease (Lambert et al., 2013^49^) | 363 | 80.6 | OR=0.99 95% CI:0.98;1.00 |
|  |  | Systolic blood pressure (Evangelou et al., 2018^66^) | Alzheimer’s Disease (Lambert et al., 2013^49^) | 343 | 77.9 | OR=1.00 95% CI:0.99;1.00 |
| **Hearing loss related exposures** | Abidin et al., (2021)^1^ | Age related hearing loss (Wells et al., 2019^69^) | Alzheimer’s Disease (Kunkle et al., 2019^51^) | 34 | NR | β=-0.26 SE=0.30 |
|  | Andrews et al., (2021)^4^ | Hearing difficulties (Wells et al., 2019^69^) | Alzheimer’s Disease (Kunkle et al., 2019^51^) | 40 | 43.1 | β=-0.13 SE=0.27 |
|  | Desai et al., (2023)^8^ | Age related hearing loss (Kalra et al., 2020)^70^ | Alzheimer’s Disease and Alzheimer’s Disease by Proxy (Bellenguez et al., 2023^52^) | 30 | 43.4 | OR=1.07 95% CI:0.85;1.33 |
|  |  | Age related hearing loss (Kalra et al., 2020)^70^ | Dementia with Lewy Bodies (Chia et al., 2021^53^) | 29 | 43.4 | OR=0.75 95% CI:0.30;1.88 |
|  |  | Age related hearing loss (Kalra et al., 2020)^70^ | Frontotemporal Dementia (Ferrari et al., 2014^54^) | 26 | 26 | OR=0.89 95% CI:0.30;2.66 |
|  | Mitchell et al., (2020)^23^ | Hearing impairment (Wells et al., 2019^69^) | Alzheimer’s Disease (Lambert et al., 2013^49^) | 35 | NR | β=0.04 SE=0.06 |
|  | Thorp et al., (2022)^35^ | Hearing impairment (Wells et al., 2019^69^) | Alzheimer’s Disease (Lambert et al., 2013^49^) | 40 | 40.9 | OR=0.89 95% CI:0.48;1.74 |
| **Obesity related exposures** | Andrews et al., (2021)^4^ | Body mass index (Yengo et al., 2018^71^) | Alzheimer’s Disease (Kunkle et al., 2019^51^) | 505 | 76.6 | β=-0.03 SE=0.045 |
|  | Chen et al., (2022)^6^ | Body mass index (MRC-IEU consortium) | Alzheimer’s Disease (Kunkle et al., 2019^51^) | 371 | 65.5 | OR=0.79 95% CI:0.58;1.10 |
|  |  | Body fat percentage (MRC-IEU consortium) | Alzheimer’s Disease (Kunkle et al., 2019^51^) | 220 | 59.9 | OR=0.62 95% CI:0.38;1.00 |
|  |  | Whole body fat-free mass (MRC-IEU consortium) | Alzheimer’s Disease (Kunkle et al., 2019^51^) | 471 | 89.1 | OR=0.79 95% CI:0.69;0.90* |
|  | Chen et al., (2023)^7^ | Body mass index (Pulit et al., 2019^72^) | Alzheimer’s Disease (Jansen et al., 2019^57^) | 472 | >10 | OR=1.04 95% CI:1.02;1.05* |
|  |  | Waist to hip ratio (Pulit et al., 2019^72^) | Alzheimer’s Disease (Jansen et al., 2019^57^) | 296 | >10 | OR=1.03 95% CI:1.01;1.05* |
|  |  | Waist to hip ratio adjusted body mass index (Pulit et al., 2019^72^) | Alzheimer’s Disease (Jansen et al., 2019^57^) | 293 | >10 | OR=0.98 95% CI:0.98;1.01 |
|  | Desai et al., (2023)^8^ | Waist to hip ratio adjusted body mass index (Pulit et al., 2019^72^) | Alzheimer’s Disease and Alzheimer’s Disease by Proxy (Bellenguez et al., 2023^52^) | 542 | 55.3 | OR=0.87 95% CI:0.82;0.96* |
|  |  | Waist to hip ratio adjusted body mass index (Pulit et al., 2019^72^) | Dementia with Lewy Bodies (Chia et al., 2021^53^) | 538 | 55.5 | OR=0.75 95% CI:0.57;0.99 |
|  |  | Waist to hip ratio adjusted body mass index (Pulit et al., 2019^72^) | Frontotemporal Dementia (Ferrari et al., 2014^54^) | 526 | 55.3 | OR=0.75 95% CI:0.58;0.97 |
|  | Huang et al., (2023)^13^ | Body mass index (Yengo et al., 2018^71^) | Alzheimer’s Disease (Kunkle et al., 2019^51^) | 715 | NR | OR=0.88 95% CI:0.83;0.94* |
|  |  | Body mass index (Yengo et al., 2018^71^) | Alzheimer’s Disease by Proxy -Maternal history (Marioni et al., 2018^55^) | 715 | NR | OR=0.92 95% CI:0.86;0.99 |
|  |  | Body mass index (Yengo et al., 2018^71^) | Alzheimer’s Disease by Proxy -Paternal history (Marioni et al., 2018^55^) | 715 | NR | OR=0.93 95% CI:0.84;1.02 |
|  | Korologou-Linden et al., (2022)^14^ | Whole body fat-free mass (UK Biobank) | Alzheimer’s Disease (Lambert et al., 2013^49^) | 521 | >30 | OR=0.79 95% CI:0.70;0.89* |
|  | Larsson et al., (2017)^15^ | Body mass index (Locke et al., 2015^73^) | Alzheimer’s Disease (Lambert et al., 2013^49^) | 76 | 17 | OR=1.05 95%C CI:0.91;1.21 |
|  |  | Waist to hip ratio adjusted body mass index (Shungin et al., 2015^74^) | Alzheimer’s Disease (Lambert et al., 2013^49^) | 38 | 17 | OR=1.18 95% CI:0.97;1.45 |
|  | Li et al., (2021)^16^ | Body mass index (Pulit et al., 2019^72^) | Alzheimer’s Disease (Jansen et al., 2019^57^) | 305 | NR | OR=1.04 95% CI:1.01;1.05* |
|  |  | Waist to hip ratio (Pulit et al., 2019^72^) | Alzheimer’s Disease (Jansen et al., 2019^57^) | 201 | NR | OR=1.02 95% CI:1.00;1.04 |
|  |  | Waist circumference (Shungin et al., 2015^74^) | Alzheimer’s Disease (Jansen et al., 2019^57^) | 38 | NR | OR=1.03 95% CI:1.00;1.07 |
|  |  | Body fat percentage (Lu et al., 2016^75^) | Alzheimer’s Disease (Jansen et al., 2019^57^) | 9 | NR | OR=1.00 95% CI:0.95;1.06 |
|  | Luo et al., (2023)^20^ | Body mass index (Yengo et al., 2018^71^) | Alzheimer’s Disease (Bellenguez et al., 2022^52^) | 440 | >10 | OR=0.94 95% CI:0.87;1.01 |
|  |  | Body mass index (Yengo et al., 2018^71^) | Alzheimer’s Disease by proxy (Bellenguez et al., 2022^52^) | 440 | >10 | OR=0.89 95% CI:0.83;0.95 |
|  | Malik et al., (2021)^21^ | Body mass index (UKBiobank) | Incident dementia (UKBiobank) | 941 | NR | OR=0.88 95% CI:0.72;1.08 |
|  | Mukerjee et al., (2015)^24^ | Body mass index (Alzheimer’s Disease Genetics Consortium) | Alzheimer’s Disease (Alzheimer’s Disease Genetics Consortium) | 31 | NR | OR=0.95 95% CI:0.90;1.01 |
|  |  | Body mass index (Health and Retirement Study) | Dementia (Health and Retirement Study) | 29 | NR | OR=1.00 95% CI:0.75;1.32 |
|  |  | Body mass index (Genetic and Environmental Risk for AD consortium) | Alzheimer’s Disease (Genetic and Environmental Risk for AD consortium) | NR | NR | OR=0.96 95% CI:0.87;1.07 |
|  | Mulugeta et al., (2021)^25^ | Unfavourable metabolic profile for body mass index (UKBiobank) | Alzheimer’s Disease (UKBiobank) | 82 | NR | OR=0.89 95% CI:0.60;1.32 |
|  |  | Unfavourable metabolic profile for body mass index (UKBiobank) | Alzheimer’s Disease (UKBiobank) | 76 | NR | OR=0.89 95% CI:0.43;1.84 |
|  | Østergaard et al., (2015)^28^ | Body mass index (Speliotes et al., 2010^76^) | Alzheimer’s Disease (Lambert et al., 2013^49^) | 49 | NR | OR=0.99 95% CI:0.80;1.19 |
|  | Thorp et al., (2022)^35^ | Body mass index (Yengo et al., 2018^71^) | Alzheimer’s Disease (Lambert et al., 2013^49^) | 418 | 74.8 | OR=0.83 95% CI:0.64;1.07 |
|  | Wang et al., (2024)^38^ | Body mass index (Howe et al., 2022^77^) | Alzheimer’s Disease (Kunkle et al., 2019^51^) | 42 | >10 | β=-0.037 95% CI:-0.078;0.004 |
|  | Zhang et al., (2020)^42^ | Body mass index (UKBiobank) | Alzheimer’s Disease (Lambert et al., 2013^49^) | 360 | NR | OR=0.90 95% CI:0.82;1.00 |
|  | Zhou et al., (2019)^44^ | Body mass index (GIANT consortium) | Alzheimer’s Disease (Lambert et al., 2013^49^) | 62 | 92.3 | OR=1.10 95% CI:0.90;1.34 |
|  |  | Waist to hip ratio (GIANT consortium) | Alzheimer’s Disease (Lambert et al., 2013^49^) | 23 | 88.1 | OR=1.05 95% CI:0.76;1.45 |
|  |  | Waist circumference (GIANT consortium) | Alzheimer’s Disease (Lambert et al., 2013^49^) | 12 | 65.5 | OR=0.97 95% CI:0.59;1.61 |
|  |  | Waist to hip ratio adjusted body mass index (GIANT consortium) | Alzheimer’s Disease (Lambert et al., 2013^49^) | 36 | 108.2 | OR=1.12 95% CI:0.89;1.41 |
|  | Zhuang et al., (2021)^47^ | Obesity (GIANT consortium) | Alzheimer’s Disease (Lambert et al., 2013^49^) | 14 | NR | OR=0.97 95% CI:0.89;1.06 |
| **Late-life** |  |  |  |  |  |  |
| **Air Pollution related exposures** | Ning et al., (2023)^26^ | Particulate Matter 2.5 (UK Biobank) | Alzheimer’s Disease (Kunkle et al., 2019^51^) | 7 | >10 | OR=1.77 95% CI:0.90;3.49 |
|  |  | Particulate Matter 10 (UK Biobank) | Alzheimer’s Disease (Kunkle et al., 2019^51^) | 19 | >10 | OR=1.93 95% CI:1.03;3.59 |
|  |  | Nitrogen Dioxide (UK Biobank) | Alzheimer’s Disease (Kunkle et al., 2019^51^) | 5 | >10 | OR=0.70 95% CI:0.24;1.98 |
|  |  | Nitrogen Oxide (UK Biobank) | Alzheimer’s Disease (Kunkle et al., 2019^51^) | 8 | >10 | OR=2.05 95% CI:0.86;4.87 |
| **Depression related exposures** | Andrews et al., (2021)^4^ | Depression (Howard et al., 2019^78^) | Alzheimer’s Disease (Kunkle et al., 2019^51^) | 84 | 43.5 | β=-0.15 SE=0.07 |
|  | Desai et al., (2023)^8^ | Depression (Howard et al., 2019^78^) | Alzheimer’s Disease and Alzheimer’s Disease by Proxy (Bellenguez et al., 2023^52^) | 76 | 42.8 | OR=1.02 95% CI:0.92;1.13 |
|  |  | Depression (Howard et al., 2019^78^) | Dementia with Lewy Bodies (Chia et al., 2021^53^) | 71 | 42.2 | OR=0.99 95% CI:0.64;1.55 |
|  |  | Depression (Howard et al., 2019^78^) | Frontotemporal Dementia (Ferrari et al., 2014^54^) | 78 | 41.9 | OR=1.00 95% CI:0.66;1.52 |
|  | Harerimana et al., (2022)^10^ | Depression (Howard et al., 2019^78^) | Alzheimer’s Disease (Jansen et al., 2019^57^) | 115 | NR | β=0.029 SE=0.01* |
|  | Hu et al., (2024)^12^ | Depression (Howard et al., 2019^78^) | Alzheimer’s Disease (GWAS ID: ieu-b-5067) | 48 | >10 | OR=1.00 95% CI:0.99;1.00 |
|  |  | Depression (Howard et al., 2019^78^) | Vascular Dementia (GWAS ID: finn-b-F5_VASCDEM) | 47 | >10 | OR=2.13 95% CI:1.25;3.64* |
|  |  | Depression (Howard et al., 2019^78^) | Parkinson’s Disease Dementia (GWAS ID: finn-b-PD_DEMENTIA) | 47 | >10 | OR=0.59 95% CI:0.20;1.72 |
|  |  | Depression (Howard et al., 2019^78^) | Dementia with Lewy Bodies (Chia et al., 2021^53^) | 44 | >10 | OR=1.01 95% CI:0.60;1.70 |
|  |  | Depression (Howard et al., 2019^78^) | Frontotemporal Dementia (Van Deerlin et al., 2010^79^) | 23 | >10 | OR=1.48 95% CI:0.47;4.63 |
|  | Thorp et al., (2022)^35^ | Depression (Howard et al., 2019^78^) | Alzheimer’s Disease (Lambert et al., 2013^49^) | 42 | 38.7 | OR=0.82 95% CI:0.67;1.01 |
| **Diabetes related exposures** | Andrews et al., (2021)^4^ | Type 2 diabetes (Xue et al., 2018^80^) | Alzheimer’s Disease (Kunkle et al., 2019^51^) | 117 | 76.7 | β=-0.02 SE=0.02 |
|  | Desai et al., (2023)^8^ | Type 2 diabetes (Scott et al., 2017^81^) | Alzheimer’s Disease and Alzheimer’s Disease by Proxy (Bellenguez et al., 2023^52^) | 39 | 62.2 | OR=1.01 95% CI:0.96;1.05 |
|  |  | Type 2 diabetes (Scott et al., 2017^81^) | Dementia with Lewy Bodies (Chia et al., 2021^53^) | 38 | 62.5 | OR=1.02 95% CI:0.86;1.22 |
|  |  | Type 2 diabetes (Scott et al., 2017^81^) | Frontotemporal Dementia (Ferrari et al., 2014^54^) | 38 | 62.5 | OR=1.02 95% CI:0.86;1.22 |
|  | Garfield et al., (2021)^9^ | Haemoglobin A1c (Wheeler et al., 2017^82^) | Alzheimer’s Disease (UK Biobank) | 51 | 164.6 | OR=1.09 95% CI:0.42;2.83 |
|  |  | Type 2 diabetes (Mahajan et al., 2018^83^) | Alzheimer’s Disease (UK Biobank) | 157 | 27.4 | OR=1.15 95% CI:0.87;1.52 |
|  | Huang et al., (2023)^13^ | Type 2 diabetes (UK Biobank) | Alzheimer’s Disease (Kunkle et al., 2019^51^) | 37 | NR | OR=0.74 95% CI:0.11;4.73 |
|  |  | Type 2 diabetes (UK Biobank) | Alzheimer’s Disease by Proxy -Maternal history (Marioni et al., 2018^55^) | 34 | NR | OR=2.73 95% CI:0.59;12.62 |
|  |  | Type 2 diabetes (UK Biobank) | Alzheimer’s Disease by Proxy -Paternal history (Marioni et al., 2018^55^) | 34 | NR | OR=0.29 95% CI:0.02;4.23 |
|  |  | Fasting Glucose (Chen et al., 2021^84^) | Alzheimer’s Disease (Kunkle et al., 2019^51^) | 64 | NR | OR=1.08 95% CI:0.84;1.38 |
|  |  | Fasting Glucose (Chen et al., 2021^84^) | Alzheimer’s Disease by Proxy -Maternal history (Marioni et al., 2018^55^) | 64 | NR | OR=0.86 95% CI:0.57;1.37 |
|  |  | Fasting Glucose (Chen et al., 2021^84^) | Alzheimer’s Disease by Proxy -Paternal history (Marioni et al., 2018^55^) | 64 | NR | OR=0.89 95% CI:0.62;1.28 |
|  |  | Fasting Insulin (Chen et al., 2021^84^) | Alzheimer’s Disease (Kunkle et al., 2019^51^) | 35 | NR | OR=1.06 95% CI:0.74;1.52 |
|  |  | Fasting Insulin (Chen et al., 2021^84^) | Alzheimer’s Disease by Proxy -Maternal history (Marioni et al., 2018^55^) | 33 | NR | OR=0.98 95% CI:0.78;1.23 |
|  |  | Fasting Insulin (Chen et al., 2021^84^) | Alzheimer’s Disease by Proxy -Paternal history (Marioni et al., 2018^55^) | 33 | NR | OR=0.82 95% CI:0.42;1.58 |
|  |  | 2 hr postprandial glucose (Chen et al., 2021^84^) | Alzheimer’s Disease (Kunkle et al., 2019^51^) | 12 | NR | OR=1.04 95% CI:0.91;1.19 |
|  |  | 2 hr postprandial glucose (Chen et al., 2021^84^) | Alzheimer’s Disease by Proxy -Maternal history (Marioni et al., 2018^55^) | 10 | NR | OR=1.04 95% CI:0.87;1.24 |
|  |  | 2 hr postprandial glucose (Chen et al., 2021^84^) | Alzheimer’s Disease by Proxy -Paternal history (Marioni et al., 2018^55^) | 10 | NR | OR=1.02 95% CI:0.80;1.30 |
|  |  | Haemoglobin A1c (Chen et al., 2021^84^) | Alzheimer’s Disease (Kunkle et al., 2019^51^) | 68 | NR | OR=1.15 95% CI:0.83;1.59 |
|  |  | Haemoglobin A1c (Chen et al., 2021^84^) | Alzheimer’s Disease by Proxy -Maternal history (Marioni et al., 2018^55^) | 66 | NR | OR=0.93 95% CI:0.68;1.25 |
|  |  | Haemoglobin A1c (Chen et al., 2021^84^) | Alzheimer’s Disease by Proxy -Paternal history (Marioni et al., 2018^55^) | 66 | NR | OR=1.09 95% CI:0.70;1.69 |
|  | Larsson et al., (2017)^15^ | Type 2 diabetes (Morris et al., 2012^85^) | Alzheimer’s Disease (Lambert et al., 2013^49^) | 50 | 62 | OR=1.02 95% CI:0.97;1.07 |
|  |  | Fasting glucose (Scott et al., 2017^81^) | Alzheimer’s Disease (Lambert et al., 2013^49^) | 36 | 72 | OR=1.14 95% CI:0.99;1.32 |
|  |  | Fasting insulin (Scott et al., 2017^81^) | Alzheimer’s Disease (Lambert et al., 2013^49^) | 19 | 34 | OR=1.13 95% CI:0.85;1.51 |
|  | Litkowski et al., (2023)^18^ | Type 2 diabetes (Mahajan et al., 2022^86^) | All Cause Dementia (European Million Veteran Cohort) | 330 | 740.4 | OR=1.07 95% CI:1.05;1.08* |
|  |  | Type 2 diabetes (Mahajan et al., 2022^86^) | Vascular Dementia (European Million Veteran Cohort) | 330 | 740.4 | OR=1.11 95% CI:1.07;1.15* |
|  |  | Type 2 diabetes (Mahajan et al., 2022^86^) | Alzheimer’s Disease (European Million Veteran Cohort) | 330 | 740.4 | OR=1.06 95% CI:1.02;1.09* |
|  |  | Type 2 diabetes (Mahajan et al., 2022^86^) | All Cause Dementia (African Million Veteran Cohort) | 330 | 57.3 | OR=1.06 95% CI:1.02;1.10* |
|  |  | Type 2 diabetes (Mahajan et al., 2022^86^) | Vascular Dementia (African Million Veteran Cohort) | 330 | 57.3 | OR=1.11 95% CI:1.04;1.19* |
|  |  | Type 2 diabetes (Mahajan et al., 2022^86^) | Alzheimer’s Disease (African Million Veteran Cohort) | 330 | 57.3 | OR=1.12 95% CI:1.02;1.23* |
|  |  | Type 2 diabetes (Mahajan et al., 2022^86^) | All Cause Dementia (Hispanic Million Veteran Cohort) | 330 | 61.8 | OR=1.04 95% CI:0.98;1.10 |
|  |  | Type 2 diabetes (Mahajan et al., 2022^86^) | Vascular Dementia (Hispanic Million Veteran Cohort) | 330 | 61.8 | OR=1.09 95% CI:0.96;1.23 |
|  |  | Type 2 diabetes (Mahajan et al., 2022^86^) | Alzheimer’s Disease (Hispanic Million Veteran Cohort) | 330 | 61.8 | OR=0.94 95% CI:0.83;1.07 |
|  | Luo et al., (2023)^20^ | Type 2 diabetes (Vujkovic et al., 2020^87^) | Alzheimer’s Disease (Bellenguez et al., 2022^52^) | 357 | >10 | OR=1.02 95% CI:0.98;1.05 |
|  |  | Type 2 diabetes (Vujkovic et al., 2020^87^) | Alzheimer’s Disease by proxy (Bellenguez et al., 2022^52^) | 353 | >10 | OR=1.00 95% CI:0.97;1.03 |
|  | Malik et al., (2021)^21^ | Haemoglobin A1c (UK Biobank) | Incident Dementia (UK Biobank) | 176 | NR | OR=1.24 95% CI: 0.82;1.88 |
|  | Meng et al., (2022)^22^ | Type 2 diabetes (Mahajan et al., 2018^83^) | Alzheimer’s Disease (Lambert et al., 2013^49^) | 37 | NR | OR=1.34 95% CI:1.05;1.70 |
|  |  | Fasting glucose (Wessel et al., 2015^88^) | Alzheimer’s Disease (Lambert et al., 2013^49^) | 7 | NR | OR=1.57 95% CI:1.14;2.17 |
|  | Østergaard et al., (2015)^28^ | Fasting glucose (Scott et al., 2017^81^) | Alzheimer’s Disease (Lambert et al., 2013^49^) | 36 | NR | OR=1.12 95% CI:0.97;1.30 |
|  |  | Insulin resistance (Scott et al., 2017^81^) | Alzheimer’s Disease (Lambert et al., 2013^49^) | 10 | NR | OR=1.32 95% CI:0.88;1.98 |
|  |  | Type 2 diabetes (Morris et al., 2012^85^) | Alzheimer’s Disease (Lambert et al., 2013^49^) | 49 | NR | OR=1.02 95% CI:0.97;1.07 |
|  | Pan et al., (2020)^30^ | Haemoglobin A1c (Wheeler et al., 2017^82^) | Alzheimer’s Disease (Lambert et al., 2013^49^) | 36 | NR | OR=0.96 95% CI:0.69;1.32 |
|  |  | Type 2 diabetes (Morris et al., 2012^85^; Scott et al., 2017^81^) | Alzheimer’s Disease (Lambert et al., 2013^49^) | 43 | NR | OR=1.02 95% CI:0.97;1.07 |
|  |  | Fasting glucose (Scott et al., 2017^81^) | Alzheimer’s Disease (Lambert et al., 2013^49^) | 28 | NR | OR=1.33 95% CI:1.04;1.68 |
|  |  | Fasting insulin (Scott et al., 2017^81^) | Alzheimer’s Disease (Lambert et al., 2013^49^) | 19 | NR | OR=1.24 95% CI:0.77;2.01 |
|  |  | Homeostasis model assessment -B-cell function (Dupuis et al., 2010^89^) | Alzheimer’s Disease (Lambert et al., 2013^49^) | 6 | NR | OR=1.92 95% CI:1.15;3.21 |
|  |  | Homeostasis model assessment -Insulin resistance (Dupuis et al., 2010^89^) | Alzheimer’s Disease (Lambert et al., 2013^49^) | 2 | NR | OR=1.17 95% CI:0.40;3.37 |
|  | Thomassen et al., (2020)^34^ | Type 2 diabetes (Scott et al., 2017^81^) | Alzheimer’s Disease (Lambert et al., 2013^49^) | 51 | NR | OR=1.04 95% CI:0.98;1.10 |
|  | Thorp et al., (2022)^35^ | Type 2 diabetes (Xue et al., 2018^80^) | Alzheimer’s Disease (Lambert et al., 2013^49^) | 111 | 22.9 | OR=1.01 95% CI:0.96;1.06 |
|  | Walter et al., (2016)^36^ | Type 2 diabetes (Morris et al., 2012^85^) | Alzheimer’s Disease (Lambert et al., 2013^49^) | 39 | NR | OR=1.01 95% CI:0.96;1.06 |
|  |  | Type 2 diabetes adiposity (Morris et al., 2012^85^) | Alzheimer’s Disease (Lambert et al., 2013^49^) | 20 | NR | OR=0.93 95% CI:0.74;1.15 |
|  |  | Type 2 diabetes beta cell function (Morris et al., 2012^85^) | Alzheimer’s Disease (Lambert et al., 2013^49^) | 9 | NR | OR=1.00 95% CI:0.94;1.07 |
|  |  | Type 2 diabetes insulin sensitivity (Morris et al., 2012^85^) | Alzheimer’s Disease (Lambert et al., 2013^49^) | 2 | NR | OR=1.17 95% CI:1.02;1.34 |
|  |  | Type 2 diabetes other biological factors (Morris et al., 2012^85^) | Alzheimer’s Disease (Lambert et al., 2013^49^) | 10 | NR | OR=0.90 95% CI:0.79;1.04 |
|  | Zhang et al., (2020)^42^ | Type 2 diabetes (Scott et al., 2017^81^) | Alzheimer’s Disease (Lambert et al., 2013^49^) | 101 | NR | OR=1.01 95% CI:0.96;0.63 |
|  |  | Fasting insulin (Scott et al., 2017^81^) | Alzheimer’s Disease (Lambert et al., 2013^49^) | 13 | NR | OR=1.50 95% CI:0.86;2.60 |
|  |  | Fasting glucose (Scott et al., 2017^81^) | Alzheimer’s Disease (Lambert et al., 2013^49^) | 32 | NR | OR=1.30 95% CI:1.01;1.66 |
|  | Zhou et al., (2019)^44^ | Fasting insulin (Scott et al., 2017^81^) | Alzheimer’s Disease (Jansen et al., 2019^57^) | 12 | 1016 | OR=1.13 95% CI:1.04;1.23* |
|  |  | Insulin resistance (Knowles et al., 2015^90^) | Alzheimer’s Disease (Jansen et al., 2019^57^) | 5 | 72 | OR=1.02 95% CI:1.00;1.03* |
|  | Xue et al., (2023)^40^ | Type 2 diabetes (Mahajan et al., 2018^83^) | Alzheimer’s Disease (Kunkle et al., 2019^51^) | 53 | >10 | OR=1.24 95% CI:1.10;1.39* |
| **Social contact related exposures** | Andrews et al., (2021)^4^ | Social isolation (Day et al., 2018^91^) | Alzheimer’s Disease (Kunkle et al., 2019^51^) | 14 | 39.0 | β=-0.42 SE=0.31 |
|  | Desai et al., (2023)^8^ | Social isolation (Day et al., 2018^91^) | Alzheimer’s Disease and Alzheimer’s Disease by Proxy (Bellenguez et al., 2023^52^) | 15 | 36.1 | OR=1.23 95% CI:0.76;1.98 |
|  |  | Social isolation (Day et al., 2018^91^) | Dementia with Lewy Bodies (Chia et al., 2021^53^) | 14 | 35 | OR=0.22 95% CI:0.03;1.47 |
|  |  | Social isolation (Day et al., 2018^91^) | Frontotemporal Dementia (Ferrari et al., 2014^54^) | 14 | 36.4 | OR=3.80 95% CI:0.68;21.30 |
|  | Shen et al., (2021)^32^ | Regular pub (Day et al., 2018^91^) | Alzheimer’s Disease (Jansen et al., 2019^57^) | 11 | 44.4 | OR=1.19 95% CI:0.98;1.44 |
|  |  | Regular pub (Day et al., 2018^91^) | Alzheimer’s Disease by proxy (Marioni et al., 2018^55^) | 11 | 44.4 | OR=0.97 95% CI:0.51;1.85 |
|  |  | Regular gym (Day et al., 2018^91^) | Alzheimer’s Disease (Jansen et al., 2019^57^) | 6 | 82.5 | OR=0.67 95% CI:0.46;0.97 |
|  |  | Regular gym (Day et al., 2018^91^) | Alzheimer’s Disease by proxy (Marioni et al., 2018^55^) | 5 | NR | OR=0.16 95% CI:0.04;0.62* |
|  |  | Regular religious group (Day et al., 2018^91^) | Alzheimer’s Disease (Jansen et al., 2019^57^) | 18 | 40.3 | OR=0.85 95% CI:0.68;1.07 |
|  |  | Regular religious group (Day et al., 2018^91^) | Alzheimer’s Disease by proxy (Marioni et al., 2018^55^) | 14 | NR | OR=0.62 95% CI:0.21;1.81 |
|  |  | Loneliness (Day et al., 2018^91^) | Alzheimer’s Disease (Jansen et al., 2019^57^) | 13 | 90.2 | OR=0.90 95% CI:0.63;1.29 |
|  |  | Loneliness (Day et al., 2018^91^) | Alzheimer’s Disease by proxy (Marioni et al., 2018^55^) | 11 | NR | OR=0.89 95% CI:0.23;3.42 |
|  |  | Loneliness (Day et al., 2018^91^) | Alzheimer’s Disease (Jansen et al., 2019^57^) | 15 | 99.1 | OR=0.99 95% CI:0.87;0.91 |
|  |  | Loneliness (Day et al., 2018^91^) | Alzheimer’s Disease by proxy (Marioni et al., 2018^55^) | 14 | 99.1 | OR=0.95 95% CI:0.66;1.37 |
|  | Thorp et al., (2022)^35^ | Loneliness (Day et al., 2018^91^) | Alzheimer’s Disease (Lambert et al., 2013^49^) | 10 | 35.9 | OR=1.12 95% CI:0.13;9.74 |
| **Physical activity related exposures** | Andrews et al., (2021)^4^ | Moderate-to-vigorous physical activity (Klimentidis et al., 2018^92^) | Alzheimer’s Disease (Kunkle et al., 2019^51^) | 20 | 33.3 | β=0.17 SE=0.25 |
|  | Baumeister et al., (2020)^5^ | Average physical activity (Klimentidis et al., 2018^92^) | Alzheimer’s Disease (Kunkle et al., 2019^51^) | 8 | >30 | OR=1.03 95% CI:0.97;1.10 |
|  |  | Vigorous physical activity (Klimentidis et al., 2018^92^) | Alzheimer’s Disease (Kunkle et al., 2019^51^) | 8 | >25 | OR=0.91 95% CI:0.46;1.81 |
|  |  | Average physical activity (Klimentidis et al., 2018^92^) | Alzheimer’s Disease (Jansen et al., 2019^57^) | 8 | >30 | OR=0.99 95% CI:0.99;1.01 |
|  |  | Vigorous physical activity (Klimentidis et al., 2018^92^) | Alzheimer’s Disease (Jansen et al., 2019^57^) | 8 | >25 | OR=1.04 95% CI:0.95;1.15 |
|  | Chen et al., (2022)^6^ | Time spent using a computer (MRC-IEU consortium) | Alzheimer’s Disease (Kunkle et al., 2019^51^) | 76 | 39 | OR=0.71 95% CI:0.52;0.99 |
|  | Desai et al., (2023)^8^ | Vigorous physical activity (Klimentidis et al., 2018^92^) | Alzheimer’s Disease and Alzheimer’s Disease by Proxy (Bellenguez et al., 2023^52^) | 7 | 38 | OR=0.48 95% CI:0.10;2.30 |
|  |  | Vigorous physical activity (Klimentidis et al., 2018^92^) | Dementia with Lewy Bodies (Chia et al., 2021^53^) | 7 | 38 | OR=0.007 95% CI:>.0001;10.45 |
|  |  | Vigorous physical activity (Klimentidis et al., 2018^92^) | Frontotemporal Dementia (Ferrari et al., 2014^54^) | 7 | 38.2 | OR=4.57 95% CI:0.12;180.71 |
|  | He et al., (2022)^17^ | Sedentary behaviours (TV watching) (van de Vegte et al., 2020^93^) | Alzheimer’s Disease (Kunkle et al., 2019^51^) | 85 | >30 | OR=1.50 95% CI:0.91;1.46 |
|  |  | Sedentary behaviours (computer use) (van de Vegte et al., 2020^93^) | Alzheimer’s Disease (Kunkle et al., 2019^51^) | 22 | >30 | OR=0.52 95% CI:0.32;0.84* |
|  |  | Sedentary behaviours (driving) (van de Vegte et al., 2020^93^) | Alzheimer’s Disease (Kunkle et al., 2019^51^) | 4 | >30 | OR=0.64 95% CI:0.22;1.92 |
|  | Korologou-Linden et al., (2022)^14^ | Self-reported moderate physical activity (UK Biobank) | Alzheimer’s Disease (Lambert et al., 2013^49^) | 14 | >30 | OR=1.40 95% CI:1.06;1.85* |
|  | Liao et al., (2022)^17^ | Fraction of accelerations >425 milli-gravities (Klimentidis et al., 2018^92^) | Alzheimer’s Disease (Kunkle et al., 2019^51^) | 2 | NR | OR=0.39 95% CI:0.08;1.98 |
|  |  | Overall acceleration average (Klimentidis et al., 2018^92^) | Alzheimer’s Disease (Kunkle et al., 2019^51^) | 8 | NR | OR=0.99 95% CI:0.77;1.27 |
|  |  | Moderate-to-vigorous physical activity (Klimentidis et al., 2018^92^) | Alzheimer’s Disease (Kunkle et al., 2019^51^) | 9 | NR | OR=0.75 95% CI:0.20;2.75 |
|  |  | Vigorous physical activity (Klimentidis et al., 2018^92^) | Alzheimer’s Disease (Kunkle et al., 2019^51^) | 5 | NR | OR=0.83 95% CI:0.08;8.53 |
|  | Malik et al., (2021)^21^ | Overall physical activity (UK Biobank) | Incident Dementia (UK Biobank) | 3 | NR | OR=1.59 95% CI:0.14;18.30 |
|  | Thorp et al., (2022)^35^ | Physical activity (Klimentidis et al., 2018^92^) | Alzheimer’s Disease (Lambert et al., 2013^49^) | 18 | 33.4 | OR=0.93 95% CI:0.52;1.66 |
|  | Wu et al., (2021)^39^ | Physical activity (Doherty et al., 2018^94^) | Alzheimer’s Disease (Kunkle et al., 2019^51^) | 5 | NR | OR=1.03 95% CI:0.48;2.21 |
|  |  | Physical activity (Klimentidis et al., 2018^92^) | Alzheimer’s Disease (Kunkle et al., 2019^51^) | 8 | NR | OR=1.03 95% CI:0.96;1.10 |
|  | Yang et al., (2021)^41^ | Sedentary behaviours (TV watching) (van de Vegte et al., 2020^93^) | Alzheimer’s Disease (Kunkle et al., 2019^51^) | NR | 5747 | OR=1.15 95% CI:0.97;1.36 |
|  |  | Sedentary behaviours (computer use) (van de Vegte et al., 2020^93^) | Alzheimer’s Disease (Kunkle et al., 2019^51^) | NR | 1685 | OR=0.67 95% CI:0.48;0.92 |
|  |  | Sedentary behaviours (driving) (van de Vegte et al., 2020^93^) | Alzheimer’s Disease (Kunkle et al., 2019^51^) | NR | 193 | OR=1.26 95% CI:0.50;3.19 |
|  | Zhang et al., (2020)^42^ | Overall activity (Doherty et al., 2018^94^) | Alzheimer’s Disease (Kunkle et al., 2019^51^) | 3 | 18.2 | OR=0.62 95% CI:0.17;2.32 |
|  |  | Sedentary behaviour (Doherty et al., 2018^94^) | Alzheimer’s Disease (Kunkle et al., 2019^51^) | 3 | 18.2 | OR=1.33 95% CI:0.68;2.60 |
|  |  | Walking (Doherty et al., 2018^94^) | Alzheimer’s Disease (Kunkle et al., 2019^51^) | 2 | 27.4 | OR=0.30 95% CI:0.13;0.68* |
|  |  | Moderate-intensity activity (Doherty et al., 2018^94^) | Alzheimer’s Disease (Kunkle et al., 2019^51^) | 25 | 2.19 | OR=0.80 95% CI:0.59;1.07 |
| **Smoking related exposures** | Andrews et al., (2021)^4^ | Cigarettes per day (Liu et al., 2019^60^) | Alzheimer’s Disease (Kunkle et al., 2019^51^) | 43 | 89.7 | β=-0.03 SE=0.15 |
|  |  | Smoking initiation (Liu et al., 2019^60^) | Alzheimer’s Disease (Kunkle et al., 2019^51^) | 342 | 51.2 | β=-0.004 SE=0.14 |
|  | Desai et al., (2023)^8^ | Lifetime smoking (Wootton et al., 2019^95^) | Alzheimer’s Disease and Alzheimer’s Disease by Proxy (Bellenguez et al., 2023^52^) | 126 | 42.7 | OR=0.80 95% CI:0.69;0.92* |
|  |  | Lifetime smoking (Wootton et al., 2019^95^) | Dementia with Lewy Bodies (Chia et al., 2021^53^) | 119 | 42.8 | OR=0.83 95% CI:0.49;1.42 |
|  |  | Lifetime smoking (Wootton et al., 2019^95^) | Frontotemporal Dementia (Ferrari et al., 2014^54^) | 123 | 88.4 | OR=0.71 95% CI:0.13;3.98 |
|  | Huang et al., (2023)^13^ | Smoking initiation (Liu et al., 2019^60^) | Alzheimer’s Disease (Kunkle et al., 2019^51^) | 73 | NR | OR=0.95 95% CI:0.81;1.13 |
|  |  | Smoking initiation (Liu et al., 2019^60^) | Alzheimer’s Disease by Proxy -Maternal history (Marioni et al., 2018^55^) | 73 | NR | OR=0.96 95% CI:0.81;1.14 |
|  |  | Smoking initiation (Liu et al., 2019^60^) | Alzheimer’s Disease by Proxy -Paternal history (Marioni et al., 2018^55^) | 73 | NR | OR=0.91 95% CI:0.68;1.23 |
|  |  | Cigarettes per day (Liu et al., 2019^60^) | Alzheimer’s Disease (Kunkle et al., 2019^51^) | 20 | NR | OR=0.96 95% CI:0.83;1.12 |
|  |  | Cigarettes per day (Liu et al., 2019^60^) | Alzheimer’s Disease by Proxy -Maternal history (Marioni et al., 2018^55^) | 20 | NR | OR=1.18 95% CI:1.01;1.37 |
|  |  | Cigarettes per day (Liu et al., 2019^60^) | Alzheimer’s Disease by Proxy -Paternal history (Marioni et al., 2018^55^) | 20 | NR | OR=1.09 95% CI:0.89;1.25 |
|  | Malik et al., (2021)^21^ | Smoking index (UK Biobank) | Incident Dementia (UK Biobank) | 126 | NR | OR=1.22 95% CI:0.77;1.94 |
|  | Larsson et al., (2017)^15^ | Smoking quantity (Thorgeirsson et al., 2010^96^) | Alzheimer’s Disease (Lambert et al., 2013^49^) | 4 | 68 | OR=0.69 95% CI:0.49;0.99 |
|  |  | Smoking initiation (Furberg et al., 2010^97^) | Alzheimer’s Disease (Lambert et al., 2013^49^) | 1 | 16 | OR=0.71 95% CI:0.37;1.33 |
|  |  | Smoking cessation (Furberg et al., 2010^97^) | Alzheimer’s Disease (Lambert et al., 2013^49^) | 1 | 103 | OR=1.16 95% CI:0.75;1.78 |
|  | Luo et al., (2023)^20^ | Smoking regularly (Liu et al., 2019^60^) | Alzheimer’s Disease (Bellenguez et al., 2022^52^) | 313 | >10 | OR=0.93 95% CI:0.87;1.00 |
|  |  | Smoking regularly (Liu et al., 2019^60^) | Alzheimer’s Disease by proxy (Bellenguez et al., 2022^52^) | 312 | >10 | OR=0.88 95% CI:0.82;0.82 |
|  | Zhang et al., (2020)^42^ | Current smoking (UK Biobank) | Alzheimer’s Disease (Lambert et al., 2013^49^) | 27 | NR | OR=0.74 95% CI:0.32;1.69 |
|  | Zhu et al., (2023)^46^ | Cigarettes per day (Matoba et al., 2019^98^) | Alzheimer’s Disease Chinese Cohort (Zhu et al., 2023^46^) | 5 | 53 | OR=0.51 95% CI:0.15;1.74 |
|  |  | Cigarettes per day (Matoba et al., 2019^98^) | Alzheimer’s Disease Japanese Cohort (Shigemizu et al., 2021^99^) | 5 | 53 | OR=1.70 95% CI:0.79;1.73 |
|  | Østergaard et al., (2015)^28^ | Smoking quantity (Furberg et al., 2010^97^) | Alzheimer’s Disease (Lambert et al., 2013^49^) | 3 | NR | OR=0.67 95% CI:0.51;0.89* |
|  |  | Smoking initiation (Furberg et al., 2010^97^) | Alzheimer’s Disease (Lambert et al., 2013^49^) | 1 | NR | OR=0.70 95% CI:0.37;1.33 |
|  | Nordesthaard et al., (2022)^27^ | Smoking cumulative (CGPS & CCHS) | All-cause Dementia (CGPS & CCHS) | 1 | 74 | OR=1.04 95% CI:0.96;1.11 |
|  |  | Smoking cumulative (CGPS & CCHS) | Alzheimer’s Disease (CGPS & CCHS) | 1 | 74 | OR=1.06 95% CI:0.97;1.16 |
|  |  | Smoking cumulative (CGPS & CCHS) | Non-Alzheimer’s dementia (CGPS & CCHS) | 1 | 74 | OR=0.98 95% CI:0.88;1.10 |
|  |  | Smoking cumulative (CGPS & CCHS) | Alzheimer’s Disease (Lambert et al., 2013^49^) | 1 | 74 | OR=0.22 95% CI:0.18;1.28 |
|  | Thorp et al., (2022)^35^ | Smoking initiation (Liu et al., 2019^60^) | Alzheimer’s Disease (Lambert et al., 2013^49^) | 77 | 41.8 | OR=0.92 95% CI:0.77;1.09 |
|  |  | Cigarettes per day (Liu et al., 2019^60^) | Alzheimer’s Disease (Lambert et al., 2013^49^) | 20 | 104 | OR=0.98 95% CI:0.83;1.15 |

GWAS: Genome Wide Association Study; IVW: Inverse variance weighted; SNP: Single Nucleotide Polymorphism; CI: Confidence Interval; OR: Odds Ratio; β: beta; SE: Standard Error; NR: Not Reported; *significant *p* value, the p-value threshold was corrected for multiple comparisons, either by using the study authors reported values if a correction had been applied or applying a Benjamini-Hochberg False Discovery Rate, ; AUDIT: Alcohol Use Disorders Identification Test; HR: Hazards Ratio; CGPS: Copenhagen General Population Study; CCHS: Copenhagen City Heart Study

Supplementary Table 2. Number and percentage of ‘high’ or ‘moderate’ quality, unique Mendelian randomization analyses per category by modifiable risk factor for clinically diagnosed dementia outcomes

| **Outcome/Modifiable risk factor** | **Robust evidence** | **Probable evidence** | **Suggestive evidence** | **Insufficient evidence** | **Total**  **evaluable** | **Non-evaluable** | **Concordant direction of effect?*** | **Direction of effect** |
| --- | --- | --- | --- | --- | --- | --- | --- | --- |
| **Alzheimer’s Disease** |  |  |  |  |  |  |  |  |
| **Early-life** |  |  |  |  |  |  |  |  |
| Education related exposures | 1 (8.3%) | 8 (66.7%) | 1 (8.3%) | 2 (16.7%) | 12 | 0 | Yes | Protective |
| **Mid-life** |  |  |  |  |  |  |  |  |
| Adiposity related exposures | 0 (0%) | 7 (36.8%) | 0 (0%) | 12 (63.2%) | 19 | 5 | No | NA |
| Alcohol consumption related exposures | 0 (0%) | 1 (11.1%) | 0 (0%) | 8 (88.9%) | 9 | 1 | No | NA |
| Blood pressure related exposures | 0 (0%) | 3 (21.4%) | 1 (7.1%) | 10 (71.4%) | 14 | 2 | No | NA |
| Hearing loss related exposures | 0 (0%) | 0 (0%) | 0 (0%) | 3 (100%) | 3 | 0 | NA | NA |
| **Late-life** |  |  |  |  |  |  |  |  |
| Depression related exposures | 0 (0%) | 0 (0%) | 0 (0%) | 2 (100%) | 2 | 1 | NA | NA |
| Diabetes related exposures | 0 (0%) | 4 (22.2%) | 1 (5.6%) | 13 (72.2%) | 18 | 4 | Yes | Risk |
| Physical activity related exposures | 2 (11.8%) | 3 (17.6%) | 0 (0%) | 12 (70.6%) | 17 | 0 | No | NA |
| Smoking related exposures | 0 (0%) | 3 (33.3%) | 0 (0%) | 6 (66.7%) | 9 | 4 | Yes | Protective |
| Social contact related exposures | 0 (0%) | 0 (0%) | 0 (0%) | 7 (100%) | 7 | 0 | NA | NA |
| **Vascular Dementia** |  |  |  |  |  |  |  |  |
| **Late-life** |  |  |  |  |  |  |  |  |
| Depression related exposures | 0 (0%) | 0 (0%) | 0 (0%) | 0 (0%) | 0 | 1 | NA | NA |
| Diabetes related exposures | 1 (33.3%) | 1(33.3%) | 0 (0%) | 1 (33.3%) | 3 | 0 | Yes | Risk |
| **Dementia with Lewy Bodies** |  |  |  |  |  |  |  |  |
| **Early-life** |  |  |  |  |  |  |  |  |
| Education related exposures | 0 (0%) | 0 (0%) | 0 (0%) | 1(100%) | 1 | 0 | NA | NA |
| **Mid-life** |  |  |  |  |  |  |  |  |
| Adiposity related exposures | 0 (0%) | 0(0%) | 0 (0%) | 1 (100%) | 1 | 0 | NA | NA |
| Alcohol consumption related exposures | 0 (0%) | 0 (0%) | 0 (0%) | 1(100%) | 1 | 0 | NA | NA |
| Blood pressure related exposures | 0 (0%) | 0 (0%) | 0 (0%) | 1(100%) | 1 | 0 | NA | NA |
| Hearing loss related exposures | 0 (0%) | 0 (0%) | 0 (0%) | 1(100%) | 1 | 0 | NA | NA |
| **Late-life** |  |  |  |  |  |  |  |  |
| Depression related exposures | 0 (0%) | 0 (0%) | 0 (0%) | 1(100%) | 1 | 0 | NA | NA |
| Diabetes related exposures | 0 (0%) | 0 (0%) | 0 (0%) | 1(100%) | 1 | 0 | NA | NA |
| Physical activity related exposures | 0 (0%) | 0 (0%) | 0 (0%) | 1(100%) | 1 | 0 | NA | NA |
| Smoking related exposures | 0 (0%) | 0 (0%) | 0 (0%) | 1(100%) | 1 | 0 | NA | NA |
| Social contact related exposures | 0 (0%) | 0 (0%) | 0 (0%) | 1(100%) | 1 | 0 | NA | NA |
| **Frontotemporal Dementia** |  |  |  |  |  |  |  |  |
| **Early-life** |  |  |  |  |  |  |  |  |
| Education related exposures | 0 (0%) | 0 (0%) | 0 (0%) | 1(100%) | 1 | 0 | NA | NA |
| **Mid-life** |  |  |  |  |  |  |  |  |
| Adiposity related exposures | 0 (0%) | 0(0%) | 0 (0%) | 1 (100%) | 1 | 0 | NA | NA |
| Alcohol consumption related exposures | 0 (0%) | 0 (0%) | 0 (0%) | 1(100%) | 1 | 0 | NA | NA |
| Blood pressure related exposures | 0 (0%) | 0 (0%) | 0 (0%) | 1(100%) | 1 | 0 | NA | NA |
| Hearing loss related exposures | 0 (0%) | 0 (0%) | 0 (0%) | 1(100%) | 1 | 0 | NA | NA |
| **Late-life** |  |  |  |  |  |  |  |  |
| Depression related exposures | 0 (0%) | 0 (0%) | 0 (0%) | 1(100%) | 1 | 1 | NA | NA |
| Diabetes related exposures | 0 (0%) | 0 (0%) | 0 (0%) | 1(100%) | 1 | 0 | NA | NA |
| Physical activity related exposures | 0 (0%) | 0 (0%) | 0 (0%) | 1(100%) | 1 | 0 | NA | NA |
| Smoking related exposures | 0 (0%) | 0 (0%) | 0 (0%) | 1(100%) | 1 | 0 | NA | NA |
| Social contact related exposures | 0 (0%) | 0 (0%) | 0 (0%) | 1(100%) | 1 | 0 | NA | NA |
| **All-Cause Dementia/Other Dementias** |  |  |  |  |  |  |  |  |
| **Mid-life** |  |  |  |  |  |  |  |  |
| Adiposity related exposures | 0 (0%) | 0 (0%) | 0 (0%) | 1(100%) | 1 | 1 | NA | NA |
| Blood pressure related exposures | 0 (0%) | 1(100%) | 0 (0%) | 0 (0%) | 1 | 0 | NA | Risk |
| **Late-life** |  |  |  |  |  |  |  |  |
| Depression related exposures | 0 (0%) | 0 (0%) | 0 (0%) | 0 (0%) | 0 | 1 | NA | NA |
| Diabetes related exposures | 1 (25%) | 1 (25%) | 1 (25%) | 1 (25%) | 4 | 0 | Yes | Risk |
| Physical activity related exposures | 0 (0%) | 0 (0%) | 0 (0%) | 0 (0%) | 0 | 1 | NA | NA |
| Smoking related exposures | 0 (0%) | 0 (0%) | 0 (0%) | 1 (100%) | 1 | 2 | NA | NA |

Robust evidence: all methods significant and in the same direction; Probable evidence: at least one main or sensitivity significant and all effects in the same direction; Suggestive evidence: at least one main or sensitivity significant but effects not all in the same direction; Insufficient evidence: all methods non-significant; Non-Evaluable: no sensitivity analysis; NA: Not applicable; *concordance assessed for estimates graded robust, probable or suggestive only.

Supplementary Table 3. Number and percentage of ‘high’ or ‘moderate’ quality, unique Mendelian randomization analyses per category by modifiable risk factor for proxy outcomes

| **Modifiable risk factor** | **Robust evidence** | **Probable evidence** | **Suggestive evidence** | **Insufficient evidence** | **Total**  **evaluable** | **Non-evaluable** | **Concordant direction of effect?*** | **Direction of effect**** |
| --- | --- | --- | --- | --- | --- | --- | --- | --- |
| **Early-life** |  |  |  |  |  |  |  |  |
| Education related exposures | 0 (0%) | 2 (66.7%) | 0 (0%) | 1 (33.33%) | 3 | 0 | Yes | Risk |
|  |  |  |  |  |  |  |  |  |
| **Mid-life** |  |  |  |  |  |  |  |  |
| Adiposity related exposures | 0 (0%) | 1 (33.3%) | 0 (0%) | 2 (66.7%) | 3 | 0 | Yes | Protective |
| Alcohol consumption related exposures | 0 (0%) | 0 (0%) | 0 (0%) | 3 (100%) | 3 | 0 | NA | NA |
| Blood pressure related exposures | 1 (10%) | 1 (10%) | 1 (10%) | 7 (70%) | 10 | 0 | No | NA |
| Hearing loss related exposures | 0 (0%) | 0 (0%) | 0 (0%) | 1 (100%) | 1 | 0 | NA | NA |
|  |  |  |  |  |  |  |  |  |
| **Late-life** |  |  |  |  |  |  |  |  |
| Depression related exposures | 0 (0%) | 0 (0%) | 0 (0%) | 1 (100%) | 1 | 0 | NA | NA |
| Diabetes related exposures | 0 (0%) | 0 (0%) | 0 (0%) | 12 (100%) | 12 | 0 | NA | NA |
| Physical activity related exposures | 0 (0%) | 0 (0%) | 0 (0%) | 1 (100%) | 1 | 0 | NA | NA |
| Smoking related exposures | 0 (0%) | 1 (20%) | 0 (0%) | 4 (80%) | 5 | 0 | NA | Protective |
| Social contact related exposures | 0 (0%) | 1 (16.7%) | 0 (0%) | 5 (83.3%) | 6 | 0 | NA | Protective |
|  |  |  |  |  |  |  |  |  |

Robust evidence: all methods significant and in the same direction; Probable evidence: at least one main or sensitivity significant and all effects in the same direction; Suggestive evidence: at least one main or sensitivity significant but effects not all in the same direction; Insufficient evidence: all methods non-significant; Non-Evaluable: no sensitivity analysis; NA: Not applicable

*concordance assessed for estimates graded robust, probable or suggestive only.

Supplementary References

1. Abidin FNZ, Wells HRR, Altmann A, Dawson SJ. Hearing difficulty is linked to Alzheimer’s disease by common genetic vulnerability, not shared genetic architecture. *npj Aging Mech Dis*. 2021;7(1). doi:10.1038/s41514-021-00069-4

2. Anderson EL, Howe LD, Wade KH, et al. Education, intelligence and Alzheimer’s disease: Evidence from a multivariable two-sample Mendelian randomization study. *Int J Epidemiol*. 2020;49(4):1163-1172. doi:10.1093/ije/dyz280

3. Andrews SJ, Goate A, Anstey KJ. Association between alcohol consumption and Alzheimer’s disease: A Mendelian randomization study. *Alzheimer’s Dement*. 2020;16(2):345-353. doi:10.1016/j.jalz.2019.09.086

4. Andrews SJ, Fulton-Howard B, O’Reilly P, et al. Causal Associations Between Modifiable Risk Factors and the Alzheimer’s Phenome. *Ann Neurol*. 2021;89(1):54-65. doi:10.1002/ana.25918

5. Baumeister SE, Karch A, Bahls M, Teumer A, Leitzmann MF, Baurecht H. Physical activity and risk of Alzheimer disease: A 2-sample mendelian randomization study. *Neurology*. 2020;95(13):E1897-E1905. doi:10.1212/WNL.0000000000010013

6. Chen SD, Zhang W, Li YZ, et al. A Phenome-wide Association and Mendelian Randomization Study for Alzheimer’s Disease: A Prospective Cohort Study of 502,493 Participants From the UK Biobank. *Biol Psychiatry*. 2022:1-12. doi:10.1016/j.biopsych.2022.08.002

7. Chen W, Feng J, Jiang S, et al. Mendelian randomization analyses identify bidirectional causal relationships of obesity with psychiatric disorders. *J Affect Disord*. 2023;339(March):807-814. doi:10.1016/j.jad.2023.07.044

8. Desai R, John A, Saunders R, et al. Examining the Lancet Commission risk factors for dementia using Mendelian randomisation. *BMJ Ment Heal*. 2023;26(1):e300555. doi:10.1136/BMJMENT-2022-300555

9. Garfield V, Farmaki AE, Fatemifar G, et al. Relationship Between Glycemia and Cognitive Function, Structural Brain Outcomes, and Dementia: A Mendelian Randomization Study in the UK Biobank. *Diabetes*. 2021;70:2313-2321.

10. Harerimana N V., Liu Y, Gerasimov ES, et al. Genetic Evidence Supporting a Causal Role of Depression in Alzheimer’s Disease. *Biol Psychiatry*. 2022;92(1):25-33. doi:10.1016/j.biopsych.2021.11.025

11. He Q, Bennett AN, Fan B, et al. Assessment of Bidirectional Relationships between Leisure Sedentary Behaviours and Neuropsychiatric Disorders: A Two-Sample Mendelian Randomization Study. *Genes (Basel)*. 2022;13(962).

12. Hu Y, Zou Y, Zhang M, Yan J, Zheng Y, Chen Y. The relationship between major depressive disorder and dementia: A bidirectional two-sample Mendelian randomization study. *J Affect Disord*. 2024;355(November 2023):167-174. doi:10.1016/j.jad.2024.03.149

13. Huang LY, Ou YN, Yang YX, Wang ZT, Tan L, Yu JT. Associations of cardiovascular risk factors and lifestyle behaviors with neurodegenerative disease: a Mendelian randomization study. *Transl Psychiatry*. 2023;13(1):1-9. doi:10.1038/s41398-023-02553-9

14. Korologou-Linden R, Bhatta L, Brumpton BM, et al. The causes and consequences of Alzheimer’s disease: phenome-wide evidence from Mendelian randomization. *Nat Commun*. 2022;13(1). doi:10.1038/s41467-022-32183-6

15. Larsson SC, Traylor M, Malik R, Dichgans M, Burgess S, Markus HS. Modifiable pathways in Alzheimer’s disease: Mendelian randomisation analysis. *BMJ*. 2017;359:5375. doi:10.1136/bmj.j5375

16. Li X, Tian Y, Yang YX, et al. Life Course Adiposity and Alzheimer’s Disease: A Mendelian Randomization Study. *J Alzheimer’s Dis*. 2021;82(2):503-512. doi:10.3233/JAD-210345

17. Liao Q, He J, Huang K. Physical activities and risk of neurodegenerative diseases: A two-sample Mendelian randomization study. *Front Aging Neurosci*. 2022;14(September):1-10. doi:10.3389/fnagi.2022.991140

18. Litkowski EM, Logue MW, Zhang R, et al. Mendelian randomization study of diabetes and dementia in the Million Veteran Program. *ALZHEIMERS Dement*. 2023;19(10):4367-4376. doi:10.1002/alz.13373

19. Liu H, Hu Y, Zhang Y, et al. Mendelian randomization highlights significant difference and genetic heterogeneity in clinically diagnosed Alzheimer’s disease GWAS and self-report proxy phenotype GWAX. *Alzheimer’s Res Ther*. 2022;14(1):1-10. doi:10.1186/s13195-022-00963-3

20. Luo J, Thomassen JQ, Bellenguez C, et al. Genetic Associations between Modifiable Risk Factors and Alzheimer Disease. *JAMA Netw Open*. 2023;6(5):1-17. doi:10.1001/jamanetworkopen.2023.13734

21. Malik R, Georgakis MK, Neitzel J, et al. Midlife vascular risk factors and risk of incident dementia: Longitudinal cohort and Mendelian randomization analyses in the UK Biobank. *Alzheimer’s Dement*. 2021;17(9):1422-1431. doi:10.1002/alz.12320

22. Meng L, Wang Z, Ji HF, Shen L. Causal association evaluation of diabetes with Alzheimer’s disease and genetic analysis of antidiabetic drugs against Alzheimer’s disease. *Cell Biosci*. 2022;12(1):1-16. doi:10.1186/s13578-022-00768-9

23. Mitchell BL, Thorp JG, Evans DM, Nyholt DR, Martin NG, Lupton MK. Exploring the genetic relationship between hearing impairment and Alzheimer’s disease. *Alzheimer’s Dement Diagnosis, Assess Dis Monit*. 2020;12(1):1-10. doi:10.1002/dad2.12108

24. Mukherjee S, Walter S, Kauwe JSK, et al. Genetically predicted body mass index and Alzheimer’s disease–related phenotypes in three large samples: Mendelian randomization analyses. *Alzheimer’s Dement*. 2015;11(12):1439-1451. doi:10.1016/J.JALZ.2015.05.015

25. Mulugeta A, Lumsden A, Hyppönen E. Unlocking the causal link of metabolically different adiposity subtypes with brain volumes and the risks of dementia and stroke: A Mendelian randomization study. *Neurobiol Aging*. 2021;102:161-169. doi:10.1016/J.NEUROBIOLAGING.2021.02.010

26. Ning P, Guo X, Qu Q, Li R. Exploring the association between air pollution and Parkinson’s disease or Alzheimer’s disease: a Mendelian randomization study. *Environ Sci Pollut Res Int*. 2023;30(59):123939-123947. doi:10.1007/s11356-023-31047-w

27. Nordestgaard AT, Nordestgaard BG, Frikke-Schmidt R, Juul Rasmussen I, Bojesen SE. Self-reported and genetically predicted coffee consumption and smoking in dementia: A Mendelian randomization study. *Atherosclerosis*. 2022;348(February):36-43. doi:10.1016/j.atherosclerosis.2022.03.022

28. Østergaard SD, Mukherjee S, Sharp SJ, et al. Associations between Potentially Modifiable Risk Factors and Alzheimer Disease: A Mendelian Randomization Study. *PLoS Med*. 2015;12(6):1-16. doi:10.1371/journal.pmed.1001841

29. Ou Y-N, Yang Y-X, Shen X-N, et al. Genetically determined blood pressure antihypertensive medications, and risk of Alzheimer’s disease: a Mendelian randomization study. *Alzheimers Res Ther*. 2021;13(41).

30. Pan Y, Chen W, Yan H, Wang M, Xiang X. Glycemic traits and Alzheimer’s disease: a Mendelian randomization study. *Aging (Albany NY)*. 2020;12(22):22688-22699. doi:10.18632/aging.103887

31. Raghavan NS, Vardarajan B, Mayeux R. Genomic variation in educational attainment modifies Alzheimer disease risk. *Neurol Genet*. 2019;5(2). doi:10.1212/NXG.0000000000000310

32. Shen LX, Yang YX, Kuo K, et al. Social Isolation, Social Interaction, and Alzheimer’s Disease: A Mendelian Randomization Study. *J Alzheimer’s Dis*. 2021;80(2):665-672. doi:10.3233/JAD-201442

33. Sproviero W, Winchester L, Newby D, et al. High Blood Pressure and Risk of Dementia: A Two-Sample Mendelian Randomization Study in the UK Biobank. *Biol Psychiatry*. 2021;89(8):817-824. doi:10.1016/j.biopsych.2020.12.015

34. Thomassen JQ, Tolstrup JS, Benn M, Frikke-Schmidt R. Type-2 diabetes and risk of dementia: Observational and Mendelian randomisation studies in 1 million individuals. *Epidemiol Psychiatr Sci*. 2020. doi:10.1017/S2045796020000347

35. Thorp JG, Mitchell BL, Gerring ZF, et al. Genetic evidence that the causal association of educational attainment with reduced risk of Alzheimer’s disease is driven by intelligence. *Neurobiol Aging*. 2022;119:127-135. doi:10.1016/j.neurobiolaging.2022.07.011

36. Walter S, Marden JR, Kubzansky LD, et al. Diabetic Phenotypes and Late Life Dementia Risk: A Mechanism-Specific Mendelian Randomization Study. *Alzheimer Dis Assoc Disord*. 2016;30(1):15-20. doi:10.1097/WAD.0000000000000128.Diabetic

37. Wang Z, Meng L, Shen L, Ji HF. Impact of modifiable risk factors on Alzheimer’s disease: A two-sample Mendelian randomization study. *Neurobiol Aging*. 2020;91:167.e11-167.e19. doi:10.1016/j.neurobiolaging.2020.02.018

38. Wang X, Chen H, Chang Z, Zhang J, Xie D. Genetic causal role of body mass index in multiple neurological diseases. *Sci Rep*. 2024;14(1):1-13. doi:10.1038/s41598-024-57260-2

39. Wu PF, Lu H, Zhou X, et al. Assessment of causal effects of physical activity on neurodegenerative diseases: A Mendelian randomization study. *J Sport Heal Sci*. 2021;10(4):454-461. doi:10.1016/j.jshs.2021.01.008

40. Xue H, Zeng L, Liu S. Unraveling the link: exploring the causal relationship between diabetes, multiple sclerosis, migraine, and Alzheimer’s disease through Mendelian randomization. *Front Neurosci*. 2023;17(August). doi:10.3389/fnins.2023.1233601

41. Yang F, Chen S, Qu Z, Wang K, Xie X, Cui H. Genetic Liability to Sedentary Behavior in Relation to Stroke, Its Subtypes and Neurodegenerative Diseases: A Mendelian Randomization Study. *Front Aging Neurosci*. 2021;13(November):1-10. doi:10.3389/fnagi.2021.757388

42. Zhang Q, Xu F, Wang L, Zhang WD, Sun CQ, Deng HW. Detecting potential causal relationship between multiple risk factors and Alzheimer’s disease using multivariable Mendelian randomization. *Aging (Albany NY)*. 2020;12(21):21747-21757. doi:10.18632/aging.103983

43. Zhang B, Huang X, Wang X, et al. Using a two-sample mendelian randomization analysis to explore the relationship between physical activity and Alzheimer’s disease. *Sci Rep*. 2022;12(1):1-9. doi:10.1038/s41598-022-17207-x

44. Zhou Y, Sun X, Zhou M. Body Shape and Alzheimer’s Disease: A Mendelian Randomization Analysis. *Front Neurosci*. 2019;13(October):1-11. doi:10.3389/fnins.2019.01084

45. Zhou M, Li H, Wang Y, Pan Y, Wang Y. Causal effect of insulin resistance on small vessel stroke and Alzheimer’s disease: A Mendelian randomization analysis. *Eur J Neurol*. 2022;29(3):698-706. doi:10.1111/ene.15190

46. Zhu Y, Guan Y, Xiao XW, et al. Mendelian randomization analyses of smoking and Alzheimer’s disease in Chinese and Japanese populations. *Front Aging Neurosci*. 2023;15. doi:10.3389/fnagi.2023.1157051

47. Zhuang QS, Meng L, Wang Z, Shen L, Ji HF. Associations Between Obesity and Alzheimer’s Disease: Multiple Bioinformatic Analyses. *J Alzheimer’s Dis*. 2021;80(1):271-281. doi:10.3233/JAD-201235

48. Okbay A, Beauchamp JP, Fontana MA, et al. Genome-wide association study identifies 74 loci associated with educational attainment. *Nature*. 2016;533(7604):539-542. doi:10.1038/nature17671

49. Lambert JC, Ibrahim-Verbaas CA, Harold D, et al. Meta-analysis of 74,046 individuals identifies 11 new susceptibility loci for Alzheimer’s disease. *Nat Genet*. 2013;45(12):1452-1458. doi:10.1038/NG.2802

50. Lee JJ, Wedow R, Okbay A, et al. Gene discovery and polygenic prediction from a genome-wide association study of educational attainment in 1.1 million individuals. *Nat Genet*. 2018;50(8):1112-1121. doi:10.1038/s41588-018-0147-3

51. Kunkle BW, Grenier-Boley B, Sims R, et al. Genetic meta-analysis of diagnosed Alzheimer’s disease identifies new risk loci and implicates Aβ, tau, immunity and lipid processing. *Nat Genet*. 2019;51(3):414-430. doi:10.1038/s41588-019-0358-2

52. Bellenguez C, Küçükali F, Jansen IE, et al. New insights into the genetic etiology of Alzheimer’s disease and related dementias. *Nat Genet*. 2022;54(4):412-436. doi:10.1038/s41588-022-01024-z

53. Chia R, Sabir MS, Bandres-Ciga S, et al. Genome sequencing analysis identifies new loci associated with Lewy body dementia and provides insights into its genetic architecture. *Nat Genet*. 2021;53(3):294-303. doi:10.1038/s41588-021-00785-3

54. Ferrari R, Hernandez DG, Nalls MA, et al. Frontotemporal dementia and its subtypes: a genome-wide association study. *Lancet Neurol*. 2014;13(7):686-699. doi:10.1016/S1474-4422(14)70065-1

55. Marioni RE, Harris SE, Zhang Q, et al. GWAS on family history of Alzheimer’s disease. *Transl Psychiatry*. 2018;8(1). doi:10.1038/s41398-018-0150-6

56. Schwartzentruber J, Cooper S, Liu JZ, et al. Genome-wide meta-analysis, fine-mapping and integrative prioritization implicate new Alzheimer’s disease risk genes. *Nat Genet*. 2021;53(3):392-402. doi:10.1038/s41588-020-00776-w

57. Jansen IE, Savage JE, Watanabe K, et al. Genome-wide meta-analysis identifies new loci and functional pathways influencing Alzheimer’s disease risk. *Nat Genet*. 2019;51(3):404-413. doi:10.1038/s41588-018-0311-9

58. Rietveld CA, Medland SE, Derringer J, et al. GWAS of 126,559 individuals identifies genetic variants associated with educational attainment. *Science (80- )*. 2013;340(6139):1467-1471. doi:10.1126/science.1235488

59. Savage JE, Jansen PR, Stringer S, et al. Genome-wide association meta-analysis in 269,867 individuals identifies new genetic and functional links to intelligence. *Nat Genet 2018 507*. 2018;50(7):912-919. doi:10.1038/s41588-018-0152-6

60. Liu M, Jiang Y, Wedow R, et al. Association studies of up to 1.2 million individuals yield new insights into the genetic etiology of tobacco and alcohol use. doi:10.1038/s41588-018-0307-5

61. Walters RK, Polimanti R, Johnson EC, et al. Transancestral GWAS of alcohol dependence reveals common genetic underpinnings with psychiatric disorders. *Nat Neurosci*. 2018;21(12):1656-1669. doi:10.1038/s41593-018-0275-1

62. Sanchez-Roige S, Palmer AA, Fontanillas P, et al. Genome-Wide Association Study Meta-Analysis of the Alcohol Use Disorders Identification Test (AUDIT) in Two Population-Based Cohorts. *Am J Psychiatry*. 2019;176:107-118. doi:10.1176/appi.ajp.2018.18040369

63. Huang KL, Marcora E, Pimenova AA, et al. A common haplotype lowers PU.1 expression in myeloid cells and delays onset of Alzheimer’s disease. *Nat Neurosci*. 2017;20(8):1052-1061. doi:10.1038/nn.4587

64. Evangelou E, Gao H, Chu C, et al. New alcohol-related genes suggest shared genetic mechanisms with neuropsychiatric disorders. *Nat Hum Behav*. 2019;3(9):950-961. doi:10.1038/s41562-019-0653-z

65. Jorgenson E, Thai KK, Hoffmann TJ, et al. Genetic contributors to variation in alcohol consumption vary by race&sol;ethnicity in a large multi-ethnic genome-wide association study. *Mol Psychiatry*. 2017;22:1359-1367. doi:10.1038/mp.2017.101

66. Evangelou E, Warren HR, Mosen-Ansorena D, et al. Genetic analysis of over 1 million people identifies 535 new loci associated with blood pressure traits. *Nat Genet*. 2018;50(10):1412-1425. doi:10.1038/s41588-018-0205-x

67. Hoffmann TJ, Ehret GB, Nandakumar P, et al. Genome-wide association analyses using electronic health records identify new loci influencing blood pressure variation. *Nat Genet*. 2017;49(1):54-64. doi:10.1038/ng.3715

68. Ehret GB, Munroe PB, Rice KM, et al. Genetic variants in novel pathways influence blood pressure and cardiovascular disease risk. *Nat 2011 4787367*. 2011;478(7367):103-109. doi:10.1038/nature10405

69. Wells HRR, Freidin MB, Zainul Abidin FN, et al. GWAS Identifies 44 Independent Associated Genomic Loci for Self-Reported Adult Hearing Difficulty in UK Biobank. *Am J Hum Genet*. 2019;105(4):788-802. doi:10.1016/J.AJHG.2019.09.008

70. Kalra G, Milon B, Casella AM, et al. Biological insights from multi-omic analysis of 31 genomic risk loci for adult hearing difficulty. *PLoS Genet*. 2020;16(9):e1009025. doi:10.1371/journal.pgen.1009025

71. Yengo L, Sidorenko J, Kemper KE, et al. Meta-analysis of genome-wide association studies for height and body mass index in ∼700 000 individuals of European ancestry. *Hum Mol Genet*. 2018;27(20):3641-3649. doi:10.1093/hmg/ddy271

72. Pulit SL, Stoneman C, Morris AP, et al. Meta-analysis of genome-wide association studies for body fat distribution in 694 649 individuals of European ancestry. *Hum Mol Genet*. 2019;28(1):166-174. doi:10.1093/HMG/DDY327

73. Locke AE, Kahali B, Berndt SI, et al. Genetic studies of body mass index yield new insights for obesity biology. *Nature*. 2015;518(7538):197-206. doi:10.1038/nature14177

74. Shungin D, Winkler T, Croteau-Chonka DC, et al. New genetic loci link adipose and insulin biology to body fat distribution. *Nature*. 2015;518(7538):187-196. doi:10.1038/nature14132

75. Lu Y, Day FR, Gustafsson S, et al. New loci for body fat percentage reveal link between adiposity and cardiometabolic disease risk. *Nat Commun 2016 71*. 2016;7(1):1-15. doi:10.1038/ncomms10495

76. Speliotes EK, Willer CJ, Berndt SI, et al. Association analyses of 249,796 individuals reveal 18 new loci associated with body mass index. 2010. doi:10.1038/ng.686

77. Howe LJ, Nivard MG, Morris TT, et al. Within-sibship genome-wide association analyses decrease bias in estimates of direct genetic effects. *Nat Genet*. 2022;54(5):581-592. doi:10.1038/s41588-022-01062-7

78. Howard DM, Adams MJ, Shirali M, et al. Genome-wide association study of depression phenotypes in UK Biobank identifies variants in excitatory synaptic pathways, April 2018. GWAS Catalog. https://www.ebi.ac.uk/gwas/publications/29662059.

79. Van Deerlin VM, Sleiman PMA, Martinez-Lage M, et al. Common variants at 7p21 are associated with frontotemporal lobar degeneration with TDP-43 inclusions. *Nat Genet*. 2010;42(3):234-239. doi:10.1038/ng.536

80. Xue A, Wu Y, Zhu Z, et al. Genome-wide association analyses identify 143 risk variants and putative regulatory mechanisms for type 2 diabetes. *Nat Commun*. 2018;9(1). doi:10.1038/s41467-018-04951-w

81. Scott RA, Scott LJ, Mägi R, et al. An Expanded Genome-Wide Association Study of Type 2 Diabetes in Europeans. *Diabetes*. 2017;66(11):2888-2902. doi:10.2337/DB16-1253/-/DC1

82. Wheeler E, Leong A, Liu CT, et al. Impact of common genetic determinants of Hemoglobin A1c on type 2 diabetes risk and diagnosis in ancestrally diverse populations: A transethnic genome-wide meta-analysis. *PLoS Med*. 2017;14(9):152. doi:10.1371/journal.pmed.1002383

83. Mahajan A, Taliun D, Thurner M, et al. Fine-mapping type 2 diabetes loci to single-variant resolution using high-density imputation and islet-specific epigenome maps. *Nat Genet 2018 5011*. 2018;50(11):1505-1513. doi:10.1038/s41588-018-0241-6

84. Chen J, Spracklen CN, Marenne G, et al. The trans-ancestral genomic architecture of glycemic traits. *Nat Genet*. 2021;53(6):840-860. doi:10.1038/s41588-021-00852-9

85. Morris AP, Voight BF, Teslovich TM, et al. Large-scale association analysis provides insights into the genetic architecture and pathophysiology of type 2 diabetes. *Nat Genet*. 2012;44(9):981-990. doi:10.1038/ng.2383

86. Mahajan A, Spracklen CN, Zhang W, et al. Multi-ancestry genetic study of type 2 diabetes highlights the power of diverse populations for discovery and translation. *Nat Genet*. 2022;54(5):560-572. doi:10.1038/s41588-022-01058-3

87. Vujkovic M, Keaton JM, Lynch JA, et al. Discovery of 318 new risk loci for type 2 diabetes and related vascular outcomes among 1.4 million participants in a multi-ancestry meta-analysis. *Nat Genet*. 2020;52(7):680-691. doi:10.1038/s41588-020-0637-y

88. Wessel J, Chu AY, Willems SM, et al. Low-frequency and rare exome chip variants associate with fasting glucose and type 2 diabetes susceptibility. *Nat Commun*. 2015;6. doi:10.1038/ncomms6897

89. Dupuis J, Langenberg C, Prokopenko I, et al. New genetic loci implicated in fasting glucose homeostasis and their impact on type 2 diabetes risk. *Nat Genet*. 2010;42(2):105-116. doi:10.1038/NG.520

90. Knowles JW, Xie W, Zhang Z, et al. Identification and validation of N-acetyltransferase 2 as an insulin sensitivity gene. *J Clin Invest*. 2015;125(4):1739-1751. doi:10.1172/JCI74692

91. Day FR, Ong KK, Perry JRB. Elucidating the genetic basis of social interaction and isolation. *Nat Commun*. 2018;9(1):1-6. doi:10.1038/s41467-018-04930-1

92. Klimentidis YC, Raichlen DA, Bea J, et al. Genome-wide association study of habitual physical activity in over 377,000 UK Biobank participants identifies multiple variants including CADM2 and APOE. *Int J Obes 2018 426*. 2018;42(6):1161-1176. doi:10.1038/s41366-018-0120-3

93. van de Vegte YJ, Said MA, Rienstra M, van der Harst P, Verweij N. Genome-wide association studies and Mendelian randomization analyses for leisure sedentary behaviours. *Nat Commun*. 2020;11(1). doi:10.1038/s41467-020-15553-w

94. Doherty A, Smith-Byrne K, Ferreira T, et al. GWAS identifies 14 loci for device-measured physical activity and sleep duration. *Nat Commun*. 2018;9(1). doi:10.1038/s41467-018-07743-4

95. Wootton RE, Richmond RC, Stuijfzand BG, et al. Evidence for causal effects of lifetime smoking on risk for depression and schizophrenia: a Mendelian randomisation study. *Psychol Med*. 2020;50(14):2435-2443. doi:10.1017/S0033291719002678

96. Thorgeirsson TE, Gudbjartsson DF, Surakka I, et al. Sequence variants at CHRNB3-CHRNA6 and CYP2A6 affect smoking behavior. *Nat Genet*. 2010;42(5):448-453. doi:10.1038/ng.573

97. Furberg H, Kim Y, Dackor J, et al. Genome-wide meta-analyses identify multiple loci associated with smoking behavior. *Nat Genet*. 2010;42(5):441-447. doi:10.1038/ng.571

98. Matoba N, Akiyama M, Ishigaki K, et al. GWAS of smoking behaviour in 165,436 Japanese people reveals seven new loci and shared genetic architecture. *Nat Hum Behav*. 2019;3(5):471-477. doi:10.1038/s41562-019-0557-y

99. Shigemizu D, Mitsumori R, Akiyama S, et al. Ethnic and trans-ethnic genome-wide association studies identify new loci influencing Japanese Alzheimer’s disease risk. *Transl Psychiatry*. 2021;11(1). doi:10.1038/s41398-021-01272-3
